# Single cobalt atoms and two oxidation states: on-surface synthesis of a 2D metal–organic framework *via* transmetallation

**DOI:** 10.1039/d6nr03391a

**Published:** 2026-09-23

**Authors:** Valentin Mischke, Olga Resel, Dominik Brandstetter, Maximilian Laßhofer, Stefania Baronio, Alessandro Namar, Sebastian Lueger, Georg Koller, Peter Puschnig, Martin Sterrer, Pierluigi Gargiani, Manuel Valvidares, Erik Vesselli, Mirko Cinchetti, Giovanni Zamborlini

**Affiliations:** a TU Dortmund University, Department of Physics 44227 Dortmund Germany; b Institute of Physics, NAWI Graz, University of Graz 8010 Graz Austria giovanni.zamborlini@uni-graz.at; c Department of Physics University of Trieste 34127 Trieste Italy; d ALBA Synchrotron Light Source 08290 Barcelona Spain; e CNR – Istituto Officina dei Materiali (IOM) 34149 Trieste Italy; f Center for Energy, Environment and Transport Giacomo Ciamician, University of Trieste 34127 Trieste Italy

## Abstract

Characterizing the chemical and physical properties of metal–organic frameworks (MOFs) is crucial for harnessing their unique functionalities in the design of next-generation catalysts and spintronic materials. Here, we report the formation of a homometallic MOF *via* co-deposition of Mn-tetrapyridylporphyrin (MnTPyP) molecules and Co atoms on an Au(100) surface. Low-energy electron diffraction and scanning tunneling microscopy measurements show that the predominant unit cell of the self-assembled MnTPyP film is commensurate with Au(100) and exhibits square symmetry, reflecting the substrate symmetry. As confirmed by scanning tunneling microscopy, Co deposition results in the formation of a long-range-ordered phase in which, as demonstrated by X-ray absorption spectroscopy, individual Co atoms coordinate the pyridyl groups of four neighboring molecules, indicating the formation of a metal–organic framework. This is accompanied by a loss of dichroism at the Mn L-edge in the X-ray absorption spectra, suggesting that the Mn atoms in the macrocycle are substituted with Co, a phenomenon known as “transmetallation”. Further X-ray photoemission measurements, supported by density functional theory calculations, reveal the presence of two non-equivalent Co species with different oxidation states: +2 for the Co in the macrocycle and +1 for the pyridyl-coordinated Co. Together, these findings highlight the critical role of the Au(100) support in promoting the transmetallation process, enabling the MOF to stabilize two non-equivalent Co centers, which can be exploited for catalytic or spintronic applications.

## Abstract 2

Characterizing the chemical and physical properties of metal–organic frameworks (MOFs) is crucial for harnessing their unique functionalities in the design of next-generation catalysts and spintronic materials.

## Graphical abstract

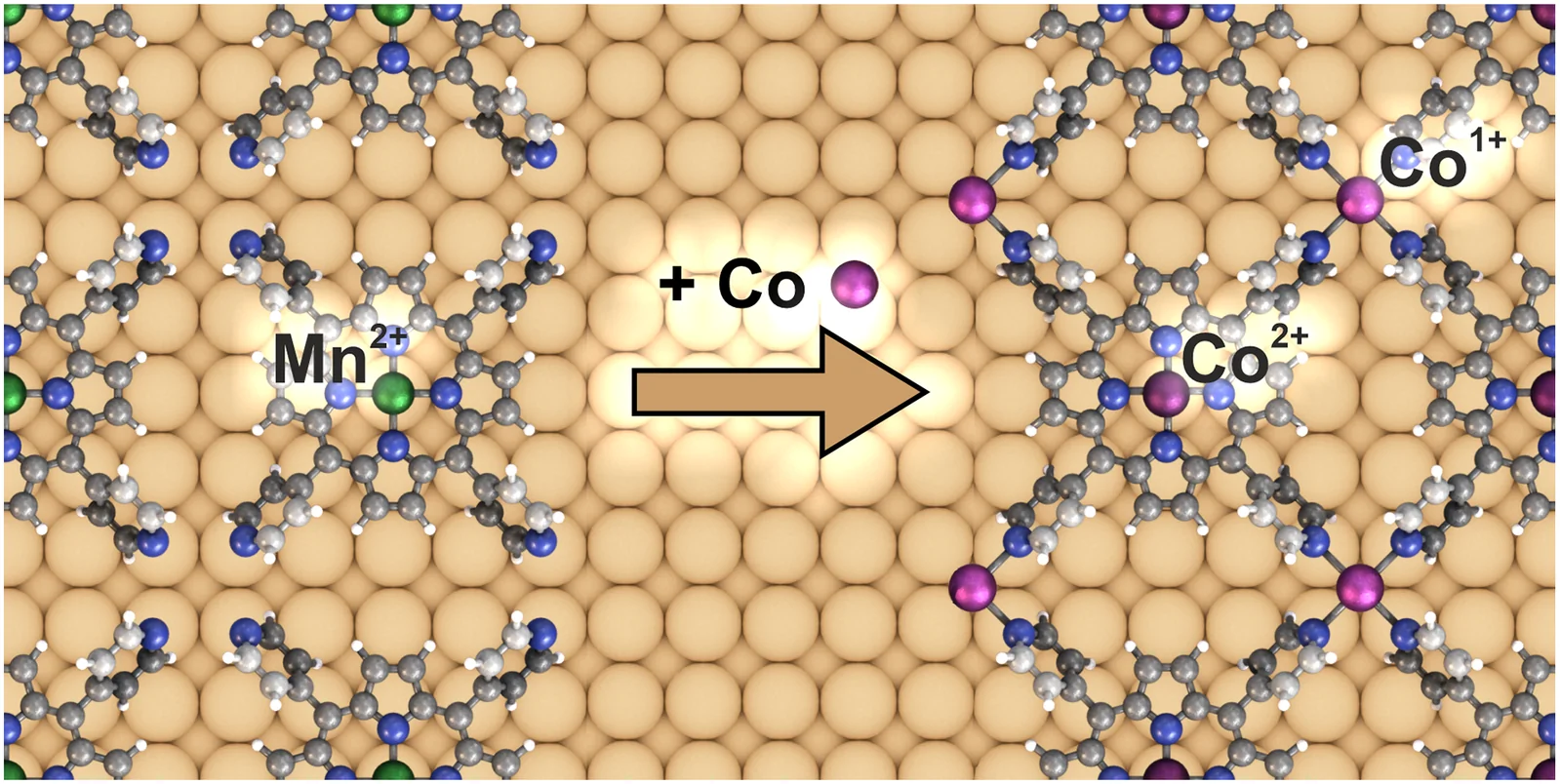

## Introduction

Metal–organic frameworks (MOFs) are a versatile class of materials that have found application in a wide range of fields, including gas sensing/storage,^1^ photocatalysis^2^ and light harvesting.^3^ MOFs consist of metal nodes (single ions or clusters) bridged by organic linkers, forming ordered and extended structures. Their modular chemistry enables exceptional surface areas, tunable pore environments and precisely designed topologies.^4^ Although the majority of MOFs come in bulk form, it is possible to synthesize surface-confined two-dimensional MOFs directly on proper substrate templates.^5^ Thanks to their high degree of crystallinity, these systems can be used as model platforms to probe intrinsic electronic^6^ and magnetic^7^ properties down to the 2D limit. On the one hand, low dimensionality introduces new challenges, as interfacial side effects arising from interactions between the building-block units and the support can no longer be neglected. On the other hand, interaction with the support can also be exploited to tune or enhance specific interfacial properties. For instance, both the reactivity and geometry of the substrate influence the structural and electronic properties of the frameworks. The Ag(100) surface, for example, can stabilize two MOF structures with a different metal-to-ligand ratio, by transferring electrons to the framework. This compensates for the charge imbalance introduced by the metal–organic coordination across the different structures.^8^ Conversely, less reactive substrates, such as graphene, only interact weakly with the MOF overlayer. This leads to an increased framework flexibility, enabling the organic linkers to adopt multiple conformations. As an example, in nickel–tetracyanoquinodimethane (Ni-TCNQ) MOFs, the TCNQ linker can undergo an axial deformation, influencing the oxidation state of the metal node.^9^

Having two metal species with different chemical environment into a surface-supported framework can further broaden the range of accessible functionalities: complementary active sites enable cooperative redox reactions,^10^ can provide bi-functionality, and may even sustain long-range ferromagnetism within the MOF.^11^ Such bimetallic frameworks can be engineered starting from metal-containing 5,10,15,20-tetra(4-pyridyl)porphyrin (M-TPyP): while one metal is already caged within the macrocycle, another one can coordinate with four pyridyl groups of adjacent molecules, forming an extended framework with a quasi-rectangular unit cell.^12–14^ These MOFs showcase single-atom coordination with a tunable electronic structure and mixed oxidation states, leading, for example, to (anti-)cooperative behavior in CO ligation.^12^ Moreover, thanks to the well-defined coordination environment, the chelated metal ion and the linking metal node can be stabilized in very specific (and different) oxidation states, as shown by the homometallic Co(i)–Co(iii)TPyP^15^ and heterometallic Co(i)–Mn(iii)TPyP MOFs^16–18^ on graphene. Notably, depending on the specific oxidation state, the metal species can become selectively reactive toward binding of small ligands^19,20^ and catalyze specific reactions at the gas–liquid or gas–solid interfaces, such as the disproportionation of nitric oxide.^21^

When it comes to on-surface synthesis, among other possible supporting substrates, graphene is extremely attractive in view of potential catalytic applications: it can endure harsh experimental conditions due to its resiliency towards oxidation. However, when growing such porphyrin-based bimetallic MOFs, graphene's hexagonal symmetry produces several rotational domains that hinder the long-range order and complicate the spectroscopic interpretation of the results.^12^ A practical route to suppress rotational degeneracy is to use supporting substrates that match the symmetry of the MOF overlayer. Since the bimetallic TPyP-based MOFs are fourfold-symmetric, any cubic single crystal surface cut along the (100) plane would provide the right template. However, to minimize the interaction between the MOF and the underlying substrate, chemically inert metals should be employed. In this regard, gold represents a suitable choice because it is expected to weakly interact with the MOF.^22^ Yet, when organometallic complexes are adsorbed on gold, the metal chelated in the tetrapyrrolic macrocycle can be exchanged with another metal atom that is subsequently deposited on the sample surface or originates from the surface itself.^23^ This process is called transmetallation, and it has been observed for several metal porphyrins and derivatives deposited on Au(111), *e.g.*, Fe → Co exchange in FeTPP^24^ and Zn → Au exchange in bis- and tetraanthracenyl Zn porphyrins.^25^ This means that, while Au(100) can help enforce a single structural domain by symmetry, gold atoms or subsequently deposited metals can swap into the framework's metal sites, altering the intended composition state during or after growth.

In this study, we synthesize a bimetallic framework on Au(100) using MnTPyP as molecular building blocks and Co atoms as metal nodes. Using a multi-technique approach that includes low-energy electron diffraction (LEED), scanning tunneling microscopy (STM), near-edge X-ray absorption spectroscopy (NEXAFS), X-ray photoelectron spectroscopy (XPS), as well as theoretical support in the form of density functional theory calculations (DFT), we investigate both the geometric and electronic properties of this 2D-MOF. On the one hand, as shown by our LEED and STM measurements, we succeed in synthesizing a MOF with a single rotational domain with square symmetry. On the other hand, at variance with the graphene substrate,^16^ our STM as well as our NEXAFS measurements at the Mn L-edge show that the Au(100) substrate promotes transmetallation at the macrocycle as Mn is replaced with Co spontaneously already at room temperature. Complementary XPS measurements of the Co 2p_3/2_ core level allow us to access the different oxidation states of the two Co species in the resulting framework: Co(ii) is stabilized in the macrocycle, as expected for standard porphyrins, whereas the other, which is coordinated with the four pyridyl moieties, is stabilized as Co(i).

## Experimental

### Structural characterization

The LEED patterns were analyzed using the “ProLEED Studio” software,^26^ which allows simulating superstructure based on user-defined matrices. First, the experimental LEED image was calibrated by using the position of the substrate spots to determine the scaling and orientation of the pattern. Next, the superstructure matrix was adjusted iteratively within the software to achieve the best agreement between the simulated and experimental LEED patterns.

The STM measurements were conducted with a commercial low-temperature scanning tunneling microscope (from CreaTec GmbH) at the University of Graz (Austria). All images were acquired with electrochemically etched tungsten tips, while the sample temperature was kept constant at 77 K (liquid nitrogen temperature). The voltage was applied to the sample.

### X-ray photoemission and absorption spectroscopy

NEXAFS measurements were acquired at the BOREAS beamline (ALBA, Barcelona, Spain). Total electron yield was performed by measuring the sample's drain current, which was normalized to that of a freshly evaporated gold grid placed in front of the sample. The incidence angle of the X-ray beam was 70° relative to the surface normal for all NEXAFS measurements, utilizing two different polarizations: s and p. The sample temperature was maintained at 300 K. A spline-like background was removed for each spectrum, independently for both polarizations. The photon energy of the nitrogen K-edge was calibrated using the drain current of the last mirror, with the first peak in absorption spectra of the adsorbed nitrogen set to 398.45 eV.^27,28^ This was only possible for the spectrum of the framework layer; for all other spectra, the first peak was set to the same energy as in the first spectrum. For the Co and Mn L-edges (using the same grating), a CoO reference sample was used, and the first peak was set to the photon energy of 776.08 eV.^29^

The XPS measurements were carried out at the University of Graz (Austria) using a Phoibos 100 spectrometer and a non-monochromatized Mg K_α_ X-ray source (both from SPECS GmbH). The spectra were referenced to the gold 4f_7/2_ peak, which was set to a binding energy of 84.0 eV.^30^ All measurements were taken in a normal emission geometry at room temperature, with an incidence angle of the X-ray beam of 55° from the surface normal. Accounting for the experimental resolution, in our fitting procedures, the lower limit of the Gaussian broadening of the peaks was set to a full width at half maximum (FWHM) of 0.58 eV and 0.86 eV, respectively, depending on the pass energy used. These values were extracted from a fit of the Au 4f core level, using identical settings for both the detector and the source. The Lorentzian width was constrained to a lower limit of 0.68 eV, due to the lifetime of the Mg transition used for the K_α_ radiation.

We would like to point out that, for the presented interface, direct XPS measurements of the Mn 2p core level shell are not feasible due to the energy overlap of both Mn 2p core level spin–orbit components (3/2 at ∼639 eV; 1/2 at ∼650 eV) with the intense signal from the Au 4p_1/2_ core level (∼643 eV). For a full MnTPyP monolayer, the Mn coverage is approximately 4% relative to the unreconstructed Au(100) surface. As a result, the signal is very weak and, in our XPS data, remains undetectable due to the predominance of the Au peak.

### Sample preparation

All samples were prepared *in situ* at different setups. First, the gold crystal surface was cleaned through cycles of sputtering with Ar^+^ ions at an energy of 2 keV, followed by annealing at 750 K. The cleanliness was verified either by the appearance of the known *c*(28 × 48) surface reconstruction^31^ in LEED (see Fig. S1), or by the absence of spectral features related to Mn, Co, N, and C in XPS and NEXAFS measurements, respectively. The commercially available Mn-*meso*-tetra(4-pyridyl)porphyrin chloride (Cl-MnTPyP) molecules (Frontier Specialty Chemicals) were degassed for several hours *in situ* at 570 K. During degassing, the Cl detaches from the molecules,^12^ as confirmed by the absence of any signature in the Cl 2p_3/2_ core level region in XPS after deposition (see SI, section II).

The MnTPyP evaporation rate was calibrated using LEED. MnTPyP was deposited stepwise onto Au(100), and LEED images were acquired after each deposition step. A nominal coverage of 1 saturated monolayer (ML) was assigned to the deposition time yielding the sharpest LEED pattern, as additional deposition beyond one monolayer resulted in blurred diffraction spots. For the XPS experiments, MnTPyP was sublimated with a Knudsen effusion cell (Kentax GmbH) at a temperature of 670 K. For the NEXAFS experiments, molecules were sublimated at 690 K, with a four cell OMBE evaporator (Dodecon Nanotechnology GmbH).

A similar procedure was used to calibrate the cobalt deposition rate on 1 ML of MnTPyP/Au(100). Co was evaporated stepwise from a rod by means of an e-beam evaporator (FOCUS GmbH) maintaining the sample temperature at around 420 K or performing post-annealing at that temperature. After each evaporation step a LEED image was acquired. We set the Co coverage that produced the sharpest LEED pattern to the nominal value of 0.08 ML (with respect to the Au surface), which corresponds to 2 Co atoms per molecule.

### Theoretical modeling

Ground-state DFT calculations for a substrate-free model of the Co–CoTPyP MOF were performed using the Vienna *Ab Initio* Simulation Package (VASP) version 6.4.3^32–37^ on the HPC facility LEONARDO hosted by Cineca.^38^ The model included only one CoTPyP molecule coordinated by a Co atom, with all coordinating pyridyls rotated in the same direction. In the *z*-direction, at least 20 Å of vacuum and a dipole layer for spurious electric fields were placed. The geometry was fully relaxed using the PBE-GGA^39^ xc-functional, until all atomic forces were below 0.01 eV Å^−1^. For the relaxation a cut-off energy of 420 eV and an effective Hubbard-*U*_eff_ parameter of 3 eV in the Dudarev ansatz^40^ as self-interaction error (SIE) correction were applied. The final density of states (DOS) calculation shown in the main text was performed with a hybrid functional (HSE06^41^), 450 eV cut-off energy and no SIE correction. A comparison to the GGA DOS shows no significant change, retrospectively validating our choice for *U*_eff_. For the geometry relaxation, the first Brillouin zone was sampled using a Γ-centered 4 × 4 × 1 grid, whereas for the DOS calculation a 6 × 6 × 1 grid was used with a reduction factor of 2 (NKREDX and NKREDY) for evaluating the exact exchange energy. All calculations were performed with a Gaussian smearing at the Fermi edge (0.05 eV) and a Grimme D3 van der Waals correction, including Becke–Johnson damping,^42,43^ where for the hybrid functional calculation a set of adjusted parameters for the HSE06 functional were used.^44^ Occupation matrix control^45^ was used only in the first steps of the geometry relaxation to ensure convergence into the correct spin configuration, with no restriction applied for neither the final part of the relaxation nor the DOS calculation.

## Results and discussion

We begin by examining the geometric properties of the pristine MnTPyP molecular film. First, the cleanliness of the Au(100) substrate was confirmed by LEED, which showed the characteristic *c*(28 × 48) reconstruction pattern (Fig. S1).^31^ Upon deposition, MnTPyP molecules form a self-assembled monolayer exhibiting long-range order, as evidenced by both LEED and STM measurements. The main results are reported in Fig. 1. LEED analysis reveals that the substrate reconstruction is lifted upon molecular deposition, and additional diffraction spots – corresponding to the long-range order of the MnTPyP film – appear in the LEED pattern, indicating a specific periodicity (marked in green, Fig. 1a). These spots are consistent with a commensurate 
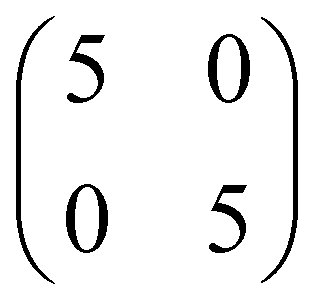
 superstructure (referenced to the unreconstructed Au(100) substrate). To further investigate the molecular arrangement, STM measurements are presented in Fig. 1b. At the selected bias voltage (+0.6 V), which lies within the bandgap of the molecule, the geometric structure is clearly resolved: the pyridyl groups appear as bright, round features, while the macrocycle exhibits a central depression corresponding to the manganese atom.^15,46^ Although an additional oblique phase was also observed (see Fig. S2b), the dominant phase is characterized by a square unit cell containing a single MnTPyP molecule. This phase forms a commensurate superstructure consistent with the observed LEED pattern. It is worth noting that, at certain bias voltages (*e.g.*, +1.4 V; see Fig. S3), the pyridyl rings no longer appear symmetric, and two molecules are then required to describe the apparent unit cell. We attribute this appearance to a purely electronic, rather than structural origin for two reasons: (i) the larger apparent unit cell is not consistent with the observed LEED pattern, and (ii) a systematic extinction of all odd-order LEED spots due to glide-plane symmetry can be excluded, since the glide planes of the apparent unit cell – which belongs to the *p*2*gg* plane group – would cause extinction of LEED spots only along the principal directions.

**Fig. 1:**
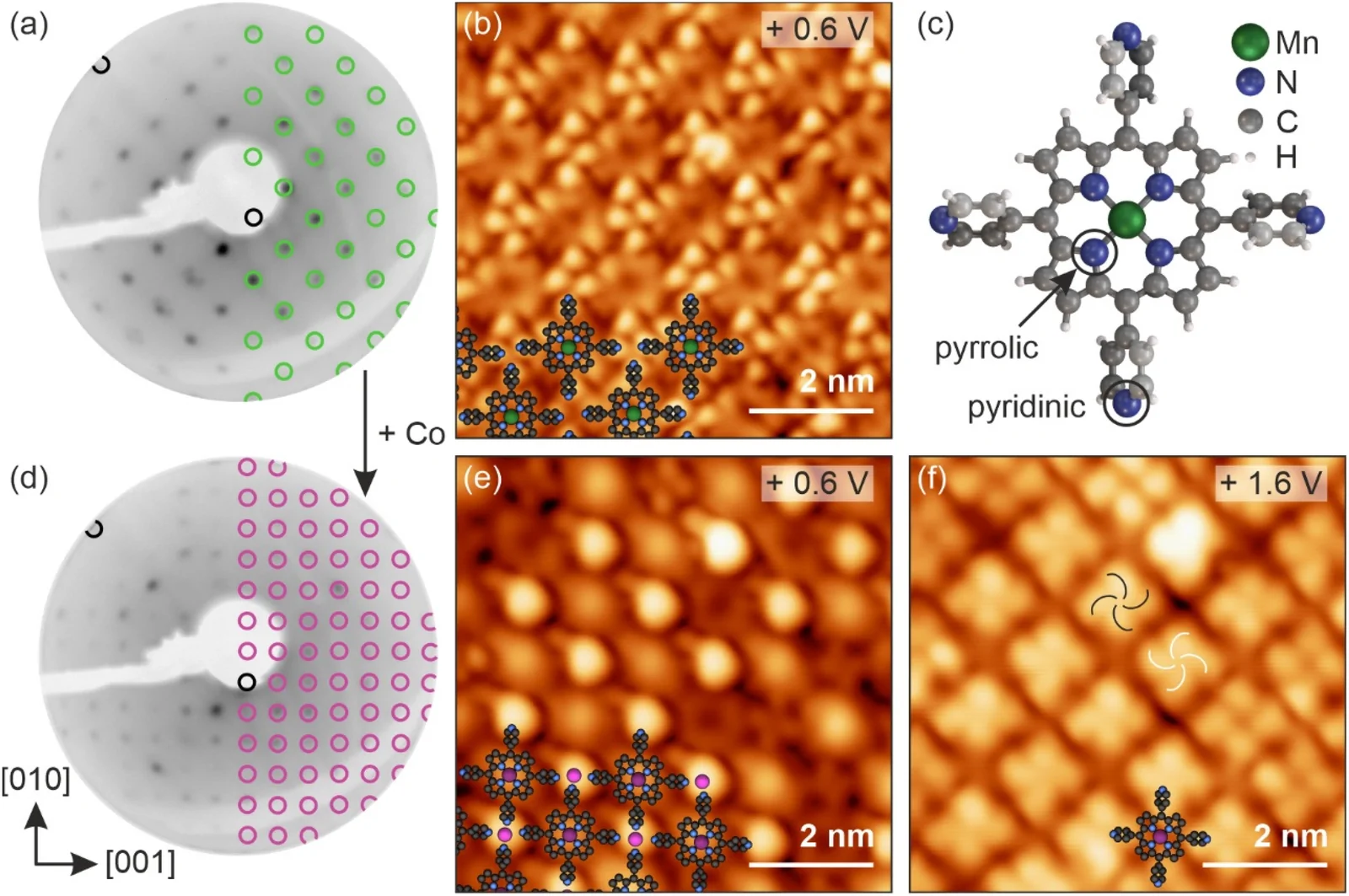
Structural characterization of the self-assembled MnTPyP layer and of the MOF. (a) LEED pattern of the self-assembled MnTPyP monolayer on Au(100). Diffraction spots corresponding to the molecular lattice are highlighted in green, while the first-order spots of the unreconstructed substrate lattice are indicated in black. (b) STM image of the self-assembled MnTPyP/Au(100) monolayer. (c) Chemical structure of a single MnTPyP molecule. (d) LEED pattern obtained after Co deposition onto MnTPyP/Au(100), leading to MOF formation. Diffraction spots associated with the MOF lattice are marked in purple. (e and f) STM image after depositing Co onto MnTPyP/Au(100). (f) Showing the alternating counterclockwise (highlighted in black) and clockwise (white) rotation of the pyridyl centers. All LEED images were acquired at an electron kinetic energy of 25 eV. All STM images were recorded at the bias voltage indicated in the figure insets, with a set tunneling current of 0.05 nA. An overlay illustrating the molecular arrangement was added to all STM images, which are 7 × 7 nm^2^. The crystallographic directions are identical for both LEED and STM and are indicated at bottom left corner of (d).

Upon cobalt deposition, both the LEED pattern and the structural features observed in STM undergo significant changes. In the LEED pattern (d) additional diffraction spots emerge, indicating the formation of a larger unit cell. Performing the same LEED analysis as for the self-assembled molecular layer yields a new superstructure described by the epitaxial matrix 
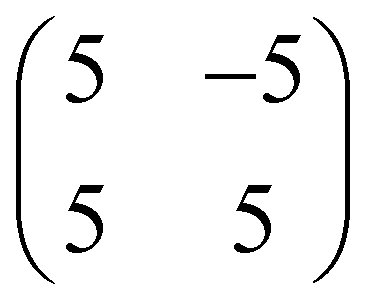
. This new unit cell has twice the area of the previous one, with its unit vectors rotated by 45° relative to the original orientation. Guided by these findings, we turn to the STM results presented in Fig. 1e to directly visualize the resulting surface structure. The STM image acquired at +0.6 V displays substantial changes: two new types of bright, round protrusions, one slightly dimmer and broader than the other, now appear at the center of the macrocycle and at the pyridyl rings, respectively. This resembles the typical appearance of Co atoms, attributed to out-of-plane 3d states that protrude into the vacuum and result in a greater apparent height.^47^ The presence of Co at the macrocycle is attributed to transmetallation,^48–50^*i.e.*, the replacement of Mn by Co – a process that will be discussed in detail in a subsequent section. In contrast, the Co atoms within the four pyridyl groups of neighboring molecules provide the first indication of MOF formation. The so-obtained structure is uniform across large areas, bigger than 100 × 100 nm^2^, as shown in Fig. S5.

Coming back to the observed doubling of the unit cell, as identified by LEED, this cannot be solely attributed to the coordination of Co atoms with the pyridyl rings, since such binding alone would not modify the lattice periodicity. When the STM images acquired at a different bias voltage are considered (namely, +1.6 V; Fig. 1f), the pyridyl groups appear to exhibit alternating clockwise and counterclockwise rotations. A similar alternating rotation has previously been reported in STM studies of Co–MnTPyP and Co–CoTPyP on graphene.^14,15^ In this configuration, all pyridyl groups coordinating to a given Co atom are rotated in the same direction. This has two consequences: (i) the fourfold symmetry of the MnTPyP molecules is broken, and (ii) neighboring molecules are azimuthally rotated by 90° with respect to one another (visualized in Fig. S6). Together, these effects give rise to a larger unit cell containing two non-equivalent molecules.

While STM suggests an interaction of the pyridyl rings with Co atoms, it does not conclusively prove that an actual coordination between Co atoms and pyridyl functional groups occurs. For this reason, we performed complementary NEXAFS measurements at the nitrogen K-edge using p- and s-polarized light, on both the pristine molecular film and the MOF. The acquired spectra are reported in Fig. 2. A key feature of NEXAFS spectroscopy is that the dichroism (*i.e.*, the polarization dependence) of the absorption intensity provides information about the geometric and electronic structure of specific molecular moieties. In particular, details on the rotation of different substituent groups and on the chemical environment of selected elements can be simultaneously obtained. These features are of special relevance in the present case, as there are two non-equivalent nitrogen species within the MnTPyP molecule: one located in the macrocycle and the other at the pyridyl functional groups, as indicated in Fig. 1c. To properly assign the different absorption resonances to specific transitions and to the corresponding nitrogen species, a thorough comparison with literature for similar systems (*e.g.*, other metalloporphyrins, pyridyl groups) was carried out. Please note that only the main resonances will be discussed in detail in the text, while a comprehensive summary of all peaks can be found in Table 1. Due to the well-known problem of absolute photon energy calibration, we compared the relative energy position of the resonances with respect to the first and most intense one (labelled as A), rather than their absolute energy positions.

**Fig. 2:**
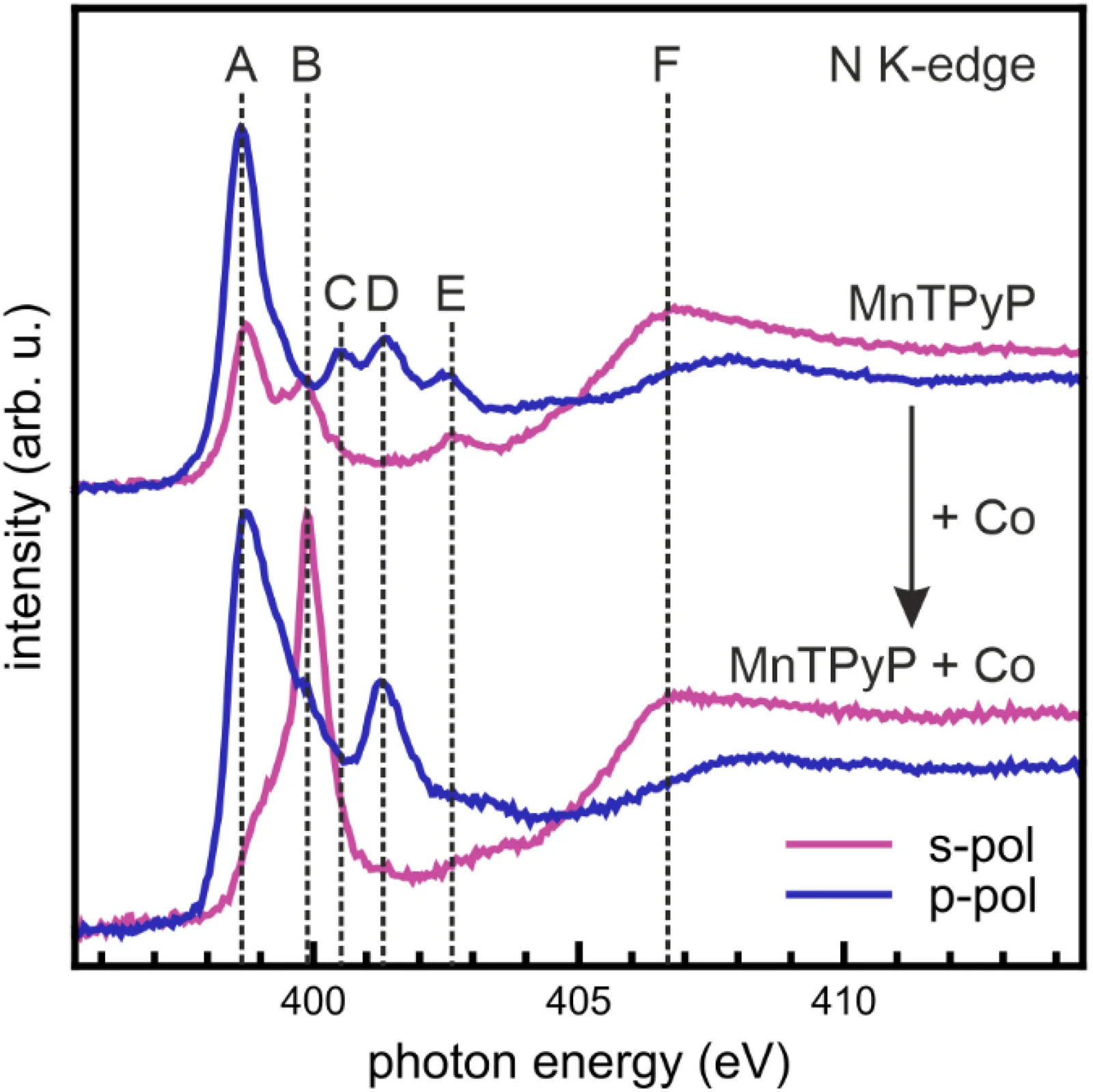
X-ray absorption measurements at the N K-edge. NEXAFS spectra of the self-assembled MnTPyP molecular layer (top) and after the deposition of Co and formation of the MOF (bottom). The measurements were performed with p- and s-polarized light, at an incident angle of 70°. The assignments of the highlighted features can be found in Table 1.

**Table 1:** Resonance assignments for the N K-edge of the pristine self-assembled molecular layer and after coordination to a framework

Peak	Energy	Assignment	Ref.
A	398.6	Macrocycle π*	15, 52, 53, 55 and 58
		Uncoordinated pyridyl π*	
B	399.8	Coordinated/adsorbed pyridyl π*	54 and 59
C	400.5	Macrocycle π* or mixed with Mn orbital	51, 57 and 60
D	401.3	Macrocycle π*	51, 52 and 60
E	402.6	Pyridyl π*	13, 54, 55 and 61
F	406.7	Macrocycle σ*	52 and 58
		Pyridyl σ*	

First, we will start discussing the pristine self-assembled MnTPyP system (Fig. 2, top). For the spectra taken with p-polarized light, we identify four sharp resonances at 398.6 eV (A), 400.5 eV (C), 401.3 eV (D) and 402.6 eV (E), along with a fifth broad *σ* resonance starting at 406.7 eV (F). Some of these resonances persist also using s-polarized light (namely, A, E and F), together with a new resonance evident at 399.7 eV (B).

Resonance D can be assigned to a transition from the N 1s states to the π*-orbitals resulting from the hybridization between the N and C atoms of the macrocycle.^51,52^ This resonance shows a strong dichroism, as its intensity nearly vanishes in s-polarization, suggesting that the macrocycle lies almost flat with respect to the surface.^52^ Peak E, instead, is attributed to the π*-orbitals of the nitrogen atoms of the pyridyl groups and shows similar intensity for both polarizations. Consequently, it becomes clear that the pyridyl moieties are rotated out of the molecular plane. This distortion is caused by the steric repulsion of the pyridyl hydrogen atoms with those at the macrocycle. For this component, we also observe a shift towards higher photon energy, compared to reported literature values, which might be related to screening effects of the Au surface.^13,53,54^ These NEXAFS findings are in agreement with the STM image in Fig. 1b: while the macrocycle appears to lie flat on the surface, the pyridyl rings stand out as bright protrusions, further supporting their rotation out of the molecular plane.

In the energy region of peak A, an overlap between the transition from the N 1s states to the π* orbitals of both the macrocycle^51,52^ and the pyridyl groups^13,55,56^ is expected. Since the energy separation between these two resonances is small (<0.15 eV),^57^ they can only be distinguished based on their different dichroic behavior. In this regard, we note that peak A is present in both polarizations. Having established (from STM as well as the dichroism of peaks D and E) that the macrocycle lies parallel to the surface, whereas the pyridyl groups are rotated, we can assign the component in p-polarization (s-polarization) to the resonances originating mostly at the macrocycle (pyridyl moiety).

Co coordination leads to shifted intensity distributions in the nitrogen K-edge spectra (Fig. 2, bottom). Now, the first peak (A) is mainly present in the p-polarized spectrum and absent in the s-polarized one. At the same time, resonance B (which is assigned to the pyridyl groups) becomes much more prominent and shows the opposite dichroism of A. This variation in lineshape cannot be attributed to conformational changes (*e.g.*, saddling of the macrocycle or rotation of the pyridyl group), which would cause only a change in dichroism of the corresponding features. It rather points to a local modification in the electronic structure of the selected nitrogen species. A small intensity on peak B was already present in the pure molecular film, attributed to a small fraction of pyridyls coordinating with the gold substrate.^62^

Since the relative intensity and dichroism of all the other resonances associated with the macrocycle remain almost unaffected by the cobalt deposition, we conclude that the pyridyl groups directly coordinate with the Co adatoms. Our interpretation is further supported by the XPS data acquired at the N 1s core level (see SI, section III). The N 1s spectrum of the pristine molecular film can be fitted with two peaks arising from the two non-equivalent nitrogen species within the MnTPyP molecule. Upon deposition of Co, the spectral contributions of both species shift towards higher binding energies. While the shift of the component associated with the nitrogen in the macrocycle (0.7 eV) is related to transmetallation, which will be discussed at a later point, the energy shift of the pyridyl peak (0.8 eV) originates from the coordination of the pyridyl groups to the Co atoms.

At this point, we want to assess the electronic properties of manganese before and after Co deposition to find final evidence for transmetallation. In this context, NEXAFS provides direct access to the chemical state of Mn, thereby enabling confirmation or disproof of the intactness of manganese macrocycles. The NEXAFS spectra acquired at the Mn L_3_-edge (transition 2p_3/2_ → 3d), before and after the deposition of Co, are presented in Fig. 3.

**Fig. 3:**
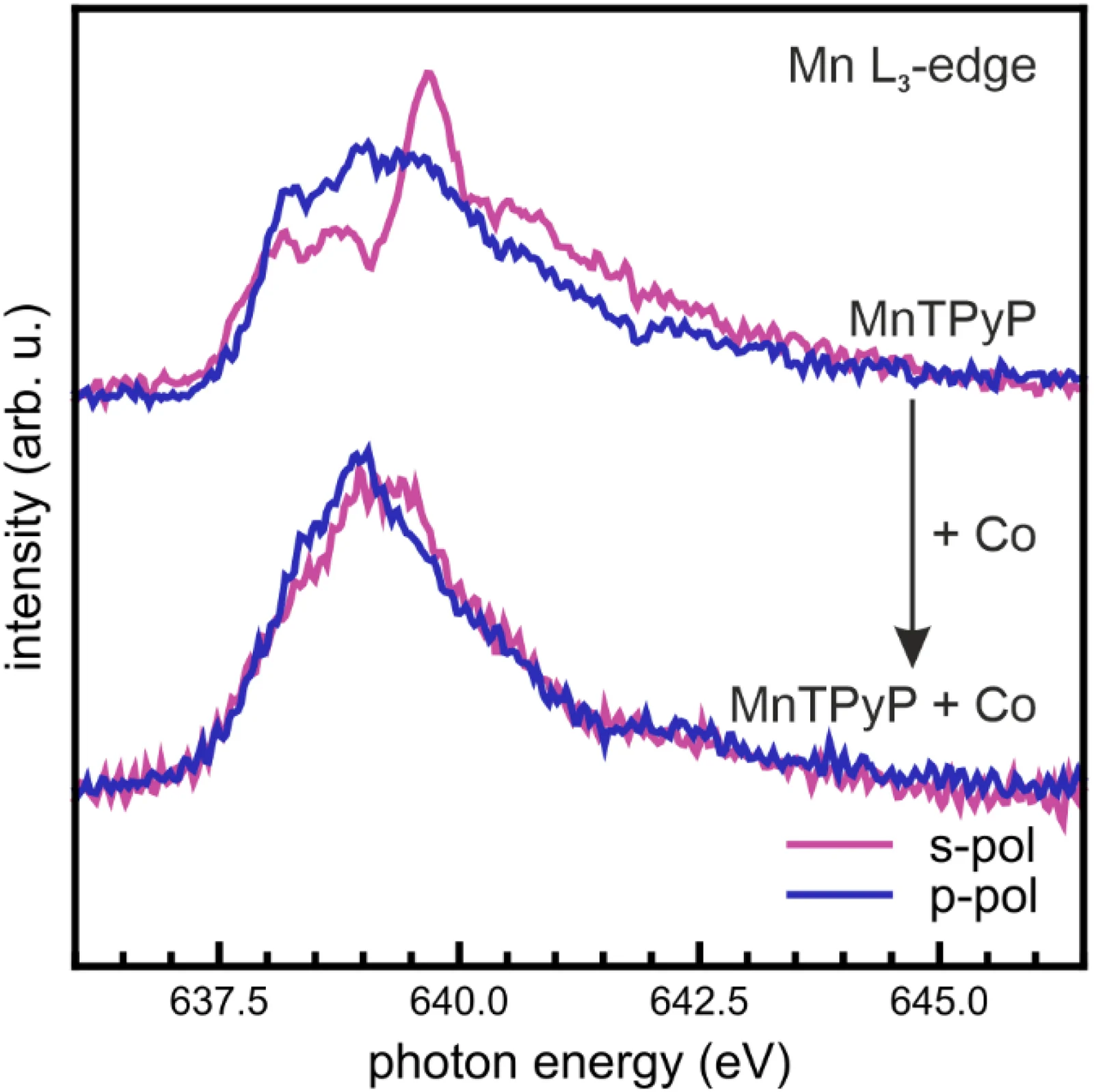
X-ray absorption measurements at the Mn L_3_-edge. NEXAFS spectra of the self-assembled MnTPyP layer (top) and of the MOF (bottom). The molecular layer shows a well-resolved multiplet structure, confirming the coordination of the Mn in the macrocycle. After Co coordination, the multiplet structure and the dichroism signal vanish, pointing to a release of the Mn from the macrocycle.

The NEXAFS spectrum of the pristine layer (Fig. 3, top) is composed of several distinguishable features that exhibit a clear dichroism. Because the porphyrin macrocycle lies parallel to the sample surface, the resonances that are enhanced in p-polarization are mainly associated with the Mn d-orbitals that have predominant out-of-plane character. Conversely, the features that are more pronounced in s-polarization are attributed to states with a stronger in-plane character. In general, for both polarizations, the fine peak structure originates from the energetic separation of the d-orbitals due to the square-planar ligand field from the coordination within the macrocycle.^24,63^ Comparison with the literature does not allow for an unambiguous determination of the actual oxidation state of Mn: it could range from +3 ^64^ to +2,^46^ or even a mixed +2/+1.^65^ However, we are keen to exclude the +3 oxidation state, since, in general, an additional ligand (*e.g.*, chloride) accepting electrons from the metal would be needed to stabilize such an oxidation state. This is the case for powder MnTPyPs, where Cl is bound to Mn and stabilizes the Cl-Mn(iii)TPyP complex (for further details, see Methods). It should be noted that, after the sublimation on Au(100), no chloride could be detected by XPS, which makes a +3 oxidation state rather unlikely (further details can be found in the SI, section II).

As anticipated from our STM findings, post-deposition of Co leads to drastic changes in the Mn L_3_-edge: in contrast to the self-assembled molecular layer, the spectra from the MOF show almost no dichroism (Fig. 3, bottom). Instead of well-separated features, only a broad peak encompassing at least three resonances appears around the photon energy of 639 eV. The absence of a linear dichroism, along with the disappearance of the multiplet splitting originating from the square-planar ligand field, indicates a bond release that reflects a non-preferred orientation of specific orbitals. Similar observations have previously been made for Fe in the case of FeTPP + Co on Au(111).^24^ Overall, this is indicative of metallic manganese on the surface,^66,67^ suggesting that the metal has been pushed out of the macrocycle. Further XPS measurements at the Co 2p_3/2_ core level, which are discussed in detail in the next section, support this finding by suggesting the occurrence of transmetallation, *i.e.*, a replacement of the Mn in the macrocycle with Co. The precise adsorption site of the Mn atoms remains unclear. Previous studies on similar systems suggest that displaced metal atoms may adsorb between the substrate and new metal centers, remain adjacent to the molecules, or form clusters on the surface.^24,49,50^ However, from our data, it is not possible to infer whether Mn forms clusters or remains close to the surface as single atoms. We would like to highlight the importance of the gold surface here: MnTPyP sublimated on graphene does not undergo transmetallation upon post-deposition of Co. In this case, it is possible to obtain a heterobimetallic organic framework, with Co atoms coordinating to the pyridyl units of adjacent molecules.^16^

Finally, transmetallation has only minor effects on the electronic structure of the macrocycle. If we now revisit the NEXAFS data acquired at the N K-edge in the case of the MOF, we notice that peak C vanishes upon coordination. Since this resonance most likely originates from the hybridization between the N and Mn orbitals, it agrees with the transmetallation scenario. In fact, it is known for similar metallophthalocyanines that the fine structure of the N K-edge depends on the chelated transition metal.^68^ Similarly, as mentioned above, the component associated with the nitrogen atoms inside the macrocycle in the N 1s core level spectrum also exhibits an energy shift upon Co deposition (see also SI, section III). More specifically, the transmetallation process affects the energy position of the pyrrole spectral contribution, which is now found at 398.6 eV, instead of 397.9 eV for the self-assembled MnTPyP layer. Comparable values have been reported for CoTPyP deposited on graphene following formation of the homometallic Co–CoTPyP MOF.^13^

Having established that in the MOF Co atoms are both in the macrocycle center of the TPyP and coordinated with the pyridyl units, we shift our focus to the chemical environment of the two different cobalt species. In this regard, both NEXAFS and XPS can be used for the identification of the oxidation states, provided that the energy distance between different Co species is wide enough. Due to the difficulty of disentangling the contributions of the different Co species in the NEXAFS Co L_3_-edge spectrum (see SI, section IV), we preferred XPS analysis, as it has proven to be more straightforward. The results of the Co 2p_3/2_ core level measurements are reported in Fig. 4. The data clearly exhibits a double peak structure. A comprehensive fit of the spectrum would have to account for the complex lineshape of Co 2p core level,^20,69–71^ due to shake-up, plasmon loss structure, and the complex multiplet splitting. However, our experimental resolution does not allow us to perform such a detailed peak analysis; thus, the spectrum was fitted with three Voigt peaks, together with a Shirley background, in order to extract the energy position of the major components that can be resolved. The fitting (for further details see SI, section V) yields a maximum intensity located at approximately 778.0 eV and 780.2 eV. The energy position of the satellite peak was fixed at 782.5 eV in agreement with the later described control experiment, as it would otherwise shift and result in a disproportionately large contribution to the fitted spectrum.

**Fig. 4:**
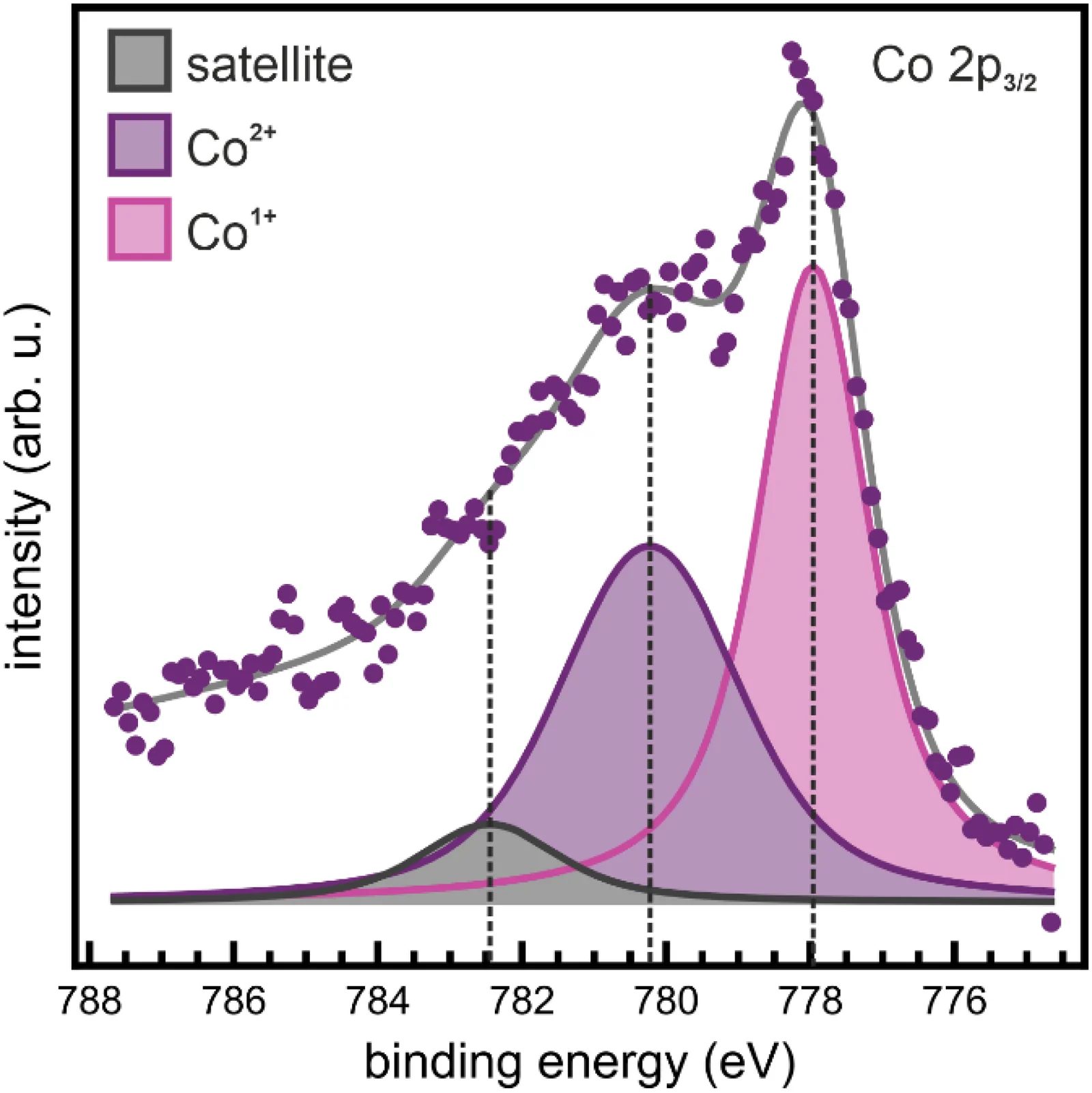
X-ray photoemission spectrum of the Co 2p_3/2_ core level. XPS spectrum at the cobalt 2p_3/2_ core level for a coverage of 0.067 ML Co atop a saturated monolayer of MnTPyP on Au(100), showing two distinguishable features. The spectrum was acquired with Mg K_α_ radiation (1254 eV) in normal emission geometry.

To assess which of the two occurring processes – transmetallation or coordination to the pyridyl substituents – is more favorable, we acquired a series of XPS spectra with increasing amounts of cobalt (see SI, section VI). As shown by these experiments, the two components grow simultaneously for higher extents of cobalt until all sites are saturated (0.067 ML Co) and metallic Co clusters start to form (0.093 ML Co). This behavior suggests that transmetallation and coordination occur in parallel, a finding that is further supported by our STM measurements.

The determination of the oxidation state, as well as the assignment of these peaks to one of the two Co adsorption sites, is based on comparison with literature and on a control experiment. In the latter, we measured the XPS spectra of the Co 2p_3/2_ core level for an as-deposited CoTPyP ML on Au(100) (*i.e.*, the final product of the transmetallation) and after the formation of the Co–CoTPyP MOF *via* Co post-deposition (for further details see SI, section V). In general, if the molecule-surface interaction is weak, as in the case of Au, it is expected that Co within the macrocycle retains the gas-phase +2 oxidation state. For a single layer of CoTPP/Au(111), this gives rise to a single peak in XPS at a binding energy of approximately 779.4 eV.^69^ Similar binding energies are also found in the case of the multilayer system, which is often used as a reference for the gas-phase molecules (*i.e.*, 779.9 eV (ref. 69) for 10 ML of CoTPP on Au). On more reactive surfaces, such as Ag and Cu, instead, the cobalt atom of the CoTPP gets reduced to the +1 oxidation state in the first molecular layer, which results in a shift of the peak towards lower binding energy.^20,70,72^ While the first CoTPP layer still interacts with the Ag surface underneath, for additional molecular layers, the molecule-surface interaction is quenched, and the +2 oxidation state is restored, with a new Co 2p_3/2_ signature located at 780 eV.^73^ Therefore, we assign the peak at 780.2 eV to the Co within the porphyrin macrocycle in the +2 oxidation state.^69,70,73^ Comparing the spectra with the ones of the pristine CoTPyP deposited on Au(100) further supports the claims, as in this case, only one component is present in the XPS Co 2p_3/2_ spectrum at 779.7 eV (See SI, section V).

The other component at 778.0 eV (Fig. 4) is associated with the Co atoms coordinated with the pyridyl groups. This agrees with our control experiment (see SI, section V), where a second peak at 778.1 eV appears only after Co deposition on the CoTPyP layer. By comparing the binding energy value with literature, we propose that the Co atom coordinated by the pyridyl moieties is in the +1 oxidation state. For example, in the CoTPP/Cu(100)^20^ system, the Co 2p_3/2_ core level shows a similar binding energy. In this case, density functional theory calculations support the scenario where the porphyrin is reduced because of charge transfer between the molecule and the substrate, stabilizing the Co(i)TPP complex. The pyridinic Co(i) species is also stabilized in the Co–CoTPyP and Co–MnTPyP MOFs grown atop of graphene,^14,16,74^ where the +1 oxidation state is also associated with an increased Co–N bond length with regard to the +2 or +3 oxidation states.^16^

However, in order to better rationalize our data, we performed density functional theory calculations for the Co–CoTPyP MOF, with the experimentally determined lattice parameters as input. Fig. 5 shows the density of states (DOS) for a substrate-free model using a hybrid exchange–correlation functional (for details see Method section) with projections onto selected frontier molecular orbitals of CoTPyP (shaded areas) and the 3d states of the pyridyl coordinated Co (purple lines). Based on earlier findings for Co porphyrins^20^ and the experimental evidence, we deliberately nudged the system to converge with the Co metal center in the macrocycle in a Co(ii) d^7^ and the coordinated Co in a Co(i) d^8^ oxidation state, using occupation matrix control. The projections onto the d-states for the Co in the macrocycle are shown in Fig. S15. Subsequently, we focused on MOF formation: our calculations reveal that the former LUMO of CoTPyP splits into singly occupied/unoccupied molecular orbitals (SOMO and SUMO, orange) upon coordination with the additional Co atom. The single integer charge transfer from the Co metal node into the molecule stabilizes cobalt in a +1 oxidation state. This interpretation is further corroborated by integrating the 3d state projections up to the Fermi level, revealing a Co(i) d^8^ for the pyridyl coordinated species alongside the Co(ii) in the macrocycle.

**Fig. 5:**
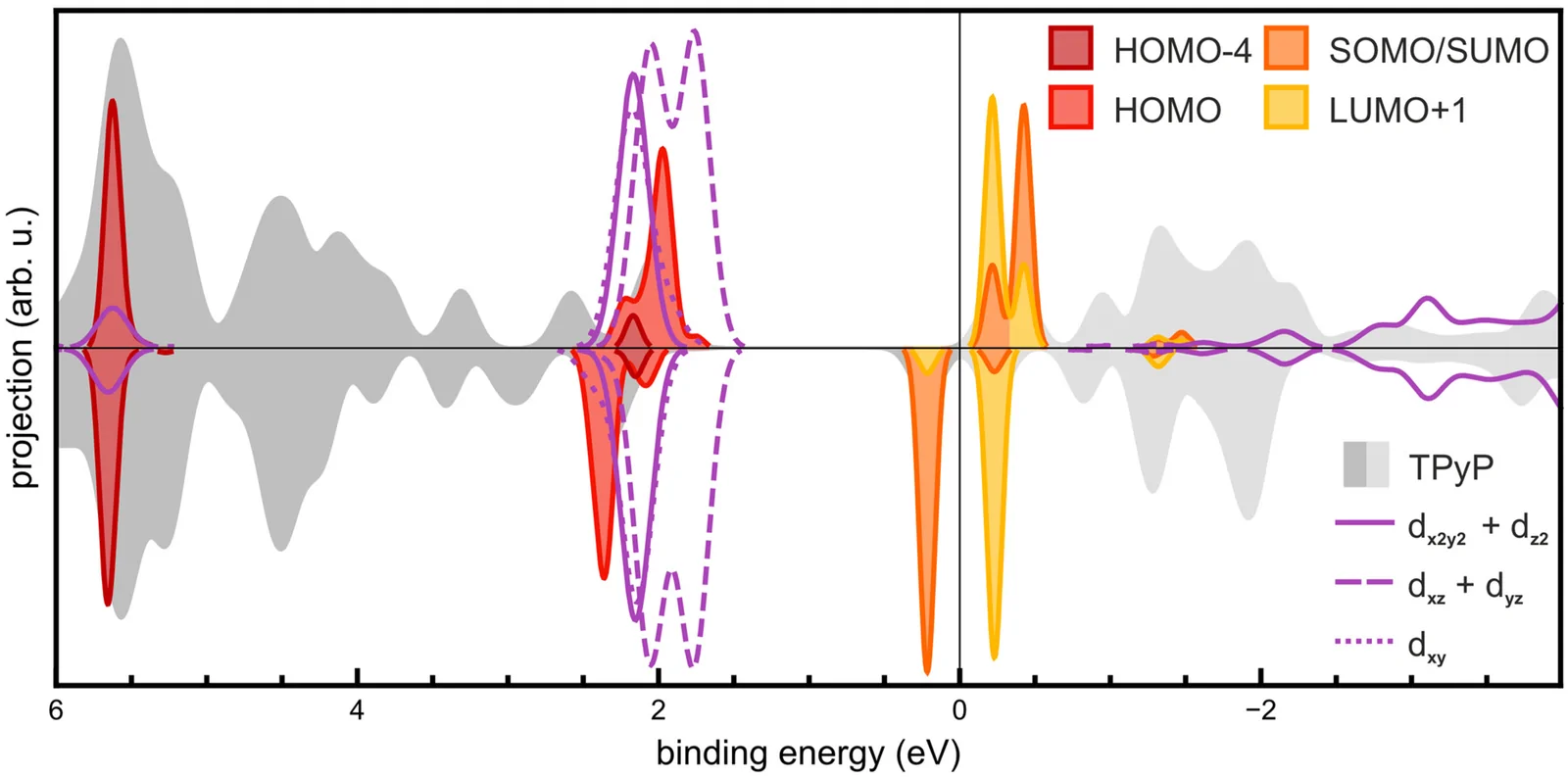
Density of states for a substrate-free transmetallated Co–CoTPyP projected onto the 3d states of the pyridyl coordinated Co (purple lines), selected frontier molecular orbitals (colored shaded areas) of CoTPyP and atomic projections onto the N, C and H atoms (gray shaded area) of the TPyP molecule.

It should be noted that the d-orbital projections displayed in Fig. 5 are defined with respect to a global coordinate frame aligned with the pyrrolic ligand axes. Hence, the projections for the pyridyl-coordinated Co atoms exhibit a mixed character owing to the locally rotated ligand coordination. This is most prevalent for the d_*x*^2^*y*^2^_ and d_*z*^2^_, nominally not degenerate in a *D*_4h_ ligand field, which is why they are depicted as one component (solid purple line). Our calculations show some degree of hybridization between this mixed d_*x*^2^*y*^2^_ + d_*z*^2^_ (solid purple line) and the HOMO−4 (dark red), apparent by the bonding/anti-bonding splitting at 5.6 eV and 2.2 eV, respectively, which can immediately be understood as σ-type bonding based on the molecular orbital shape. Additionally, the d_*x*^2^*y*^2^_ + d_*z*^2^_ combination shows a very dispersive character above Fermi, spanning several electronvolts from about −1.5 eV to −4.2 eV, with consistent overlap with the molecular projections onto the CoTPyP, further indicating a long-range Co–CoTPyP interaction and formation of a metal–organic framework. To summarize, our DFT simulations support the assignment of a mixed +1 and +2 oxidation state for the Co coordinated by the pyridyl rings and Co chelated in the macrocycle, respectively. At the same time, they also exhibit hybridization between the d-states of the Co metal nodes and the porphyrin frontier orbitals, characteristic of MOF formation.

## Conclusion

In this paper, we report the synthesis of a 2D MOF on a Au(100) surface, constructed from MnTPyP molecular building blocks and cobalt metal nodes. The self-assembled MnTPyP film and the MOF obtained after Co deposition were investigated using a multi-technique approach comprising LEED, STM, NEXAFS, and XPS. The pristine MnTPyP film is arranged in a square unit cell, lifting the surface reconstruction. STM and NEXAFS measurements reveal that the macrocycle lies flat on the surface, while the pyridyl moieties are rotated out of the molecular plane. X-ray absorption spectra exhibit a pronounced linear dichroism at the Mn L_3_-edge. Based on the lineshape and energy position of the resonances, we deduce a +2 oxidation state for the Mn atom. Upon cobalt deposition, STM measurements show that new bright protrusions, associated with the Co atoms, appear between the pyridyl rings and at the center of the macrocycle. This anticipates our spectroscopic findings: (i) the shift observed in the NEXAFS K-edge spectra, which confirms the formation of a MOF through cobalt coordination to the pyridinic nitrogen atoms, (ii) the absence of dichroic signal at the L_3_-edge of Mn, which is a fingerprint of transmetallation. Owing to the symmetry match between the MOF and the Au(100) substrate, the resulting homometallic Co–CoTPyP framework exhibits a single rotational domain. The two Co species are non-equivalent, exhibiting different oxidation states: +2 for Co within the macrocycle and +1 for Co coordinated to the pyridyl moieties, as confirmed by XPS and DFT.

Our results also highlight the pivotal role of the substrate in determining the geometrical and electronic properties of the MOF. While the substrate symmetry allows for the formation of a single rotational domain, the surface also promotes metal-ion exchange within the porphyrin. These findings are particularly relevant for the fabrication of well-defined MOF/metal interfaces, with potential applications in areas such as molecular spintronics and catalysis, with broad implications wherever the oxidation state of the single metal atom governs the chemical or physical properties of the system. Moreover, the presence of two non-equivalent single Co atoms with different oxidation state in the MOF opens the way towards bi-functionality in a mono-phasic material, which is particularly attractive for secondary batteries electrodes chemistry.

## Author contributions

Conceptualization, V. M. and G. Z.; methodology, experimental investigation, V. M., O. R., G. Z.; experimental support, M. L., A. N., S. B., S. L., G. K., M. S., P. G., M. V.; simulation. D. B, P. P.; data analysis. V. M., O. R.; writing – original draft, V. M. and G. Z.; writing – review and editing, V. M., G. Z., O. R., D. B.; funding acquisition, G. Z., E. V., P. P. and M. C. All authors revised and discussed the final version of the manuscript.

## Conflicts of interest

The authors declare no conflicts of interest.

## Supplementary Material

NR-018-D6NR03391A-s001

## Data Availability

The data underpinning our findings are available from the authors upon reasonable request. Supplementary information (SI) is available. See DOI: https://doi.org/10.1039/d6nr03391a.
