# Supplementary material for: Single cobalt atoms and two oxidation states: on-surface synthesis of a 2D metal–organic framework via transmetallation
Source: Nanoscale. 2026 Sep 23;18(40):22160–72. doi: 10.1039/d6nr03391a (PMC13632671; doi:10.1039/d6nr03391a)
Supplement: Supplementary file 1 [file NR-018-D6NR03391A-s001.pdf]

Supplementary Information (SI) for Nanoscale.
This journal is © The Royal Society of Chemistry

Supplementary information of: "Single cobalt atoms and two oxidation states: on-surface synthesis of a 2D metal-organic framework via transmetallation"

Valentin Mischke,[1,#] Olga Resel,[2,#] Dominik Brandstetter,[2] Maximilian Laßhofer,[2] Stefania Baronio,[3] Alessandro Namar,[3] Sebastian Lueger,[2] Georg Koller,[2] Peter Puschnig,[2] Martin Sterrer,[2] Pierluigi Gargiani,[4] Manuel Valvidares,[4] Erik Vesselli,[3,5,6] Mirko Cinchetti,[1] Giovanni Zamborlini[1,2,*]

[1] TU Dortmund University, Department of Physics, 44227 Dortmund, Germany
[2] Institute of Physics, NAWI Graz, University of Graz, 8010 Graz, Austria
[3] Department of Physics University of Trieste, 34127 Trieste, Italy
[4] ALBA Synchrotron Light Source, 08290 Barcelona, Spain
[5]CNR – Istituto Officina dei Materiali (IOM), 34149 Trieste, Italy
[6]Center for Energy, Environment and Transport Giacomo Ciamician, University of Trieste, 34127 Trieste, Italy
[#]these authors contributed equally to this work
*corresponding author: giovanni.zamborlini@uni-graz.at

Section I – Structure

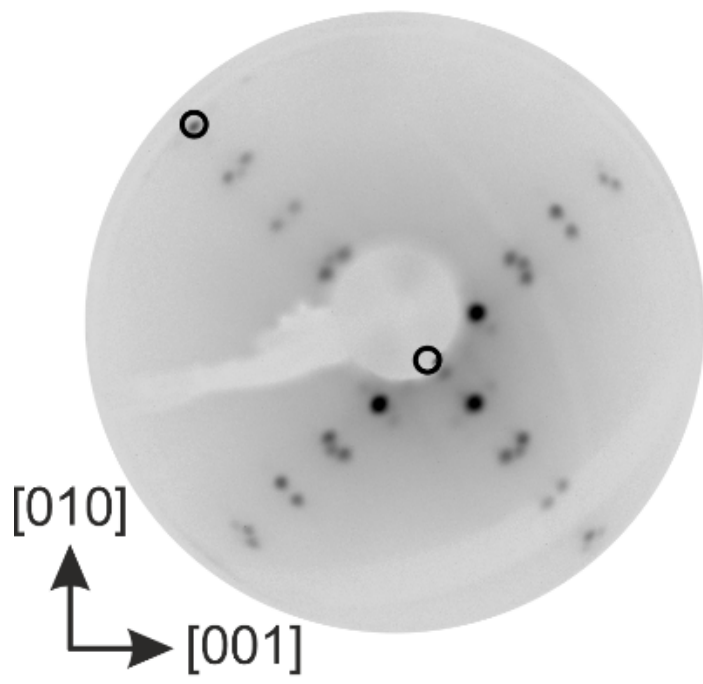

Figure S1. LEED pattern of clean Au(100) acquired at 25 eV. First order diffraction spots of the unreconstructed lattice are marked in black.

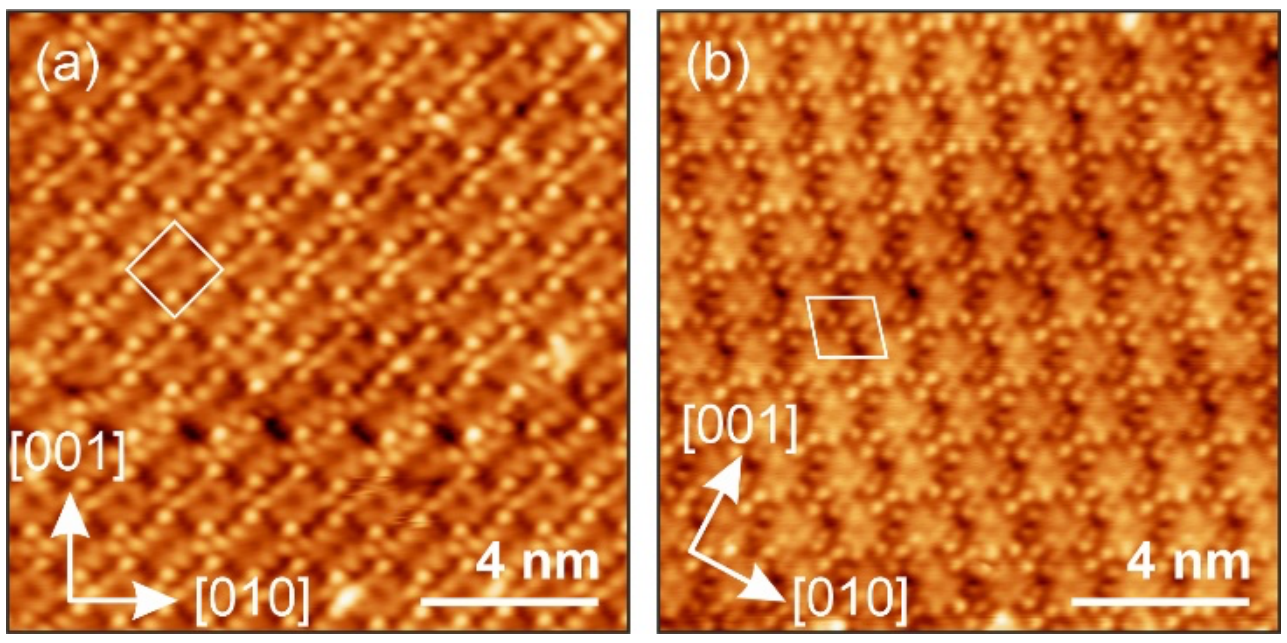

Figure S2. Large-scale STM images of self-assembled MnTPyP on Au(100). (a) The dominant phase, with a unit cell, as determined by LEED, indicated by a white square. (b) An additional phase observed in STM, with an oblique unit cell outlined by a white parallelogram. All images were acquired at a bias voltage of + 0.6 V with a set tunneling current of 0.05 nA. Image size 14 x 14 nm^2.

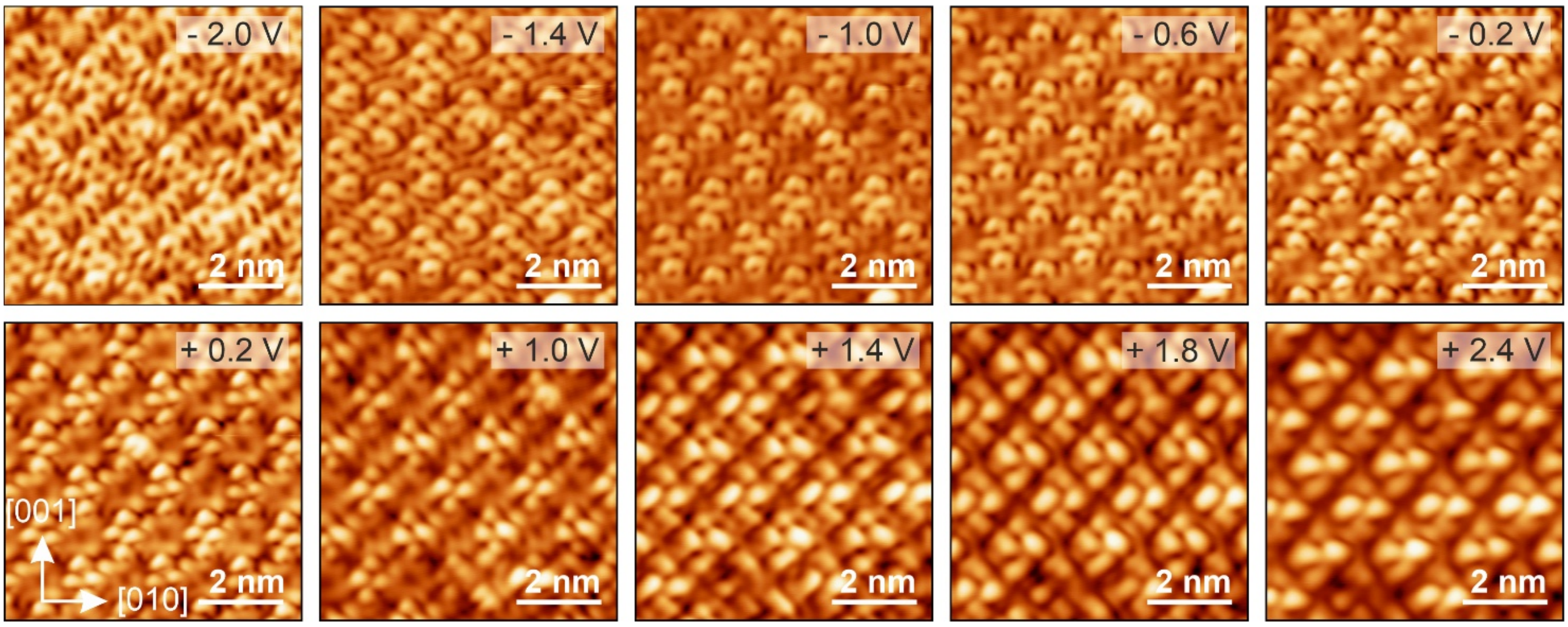

Figure S3. STM images of self-assembled MnTPyP on Au(100), acquired at different bias voltages as indicated by the insets. All images were recorded with a set tunneling current of 0.05 nA. The image size for each measurement is 7 x 7 nm^2. The crystallographic directions for all images are indicated by arrows in the bottom left corner.

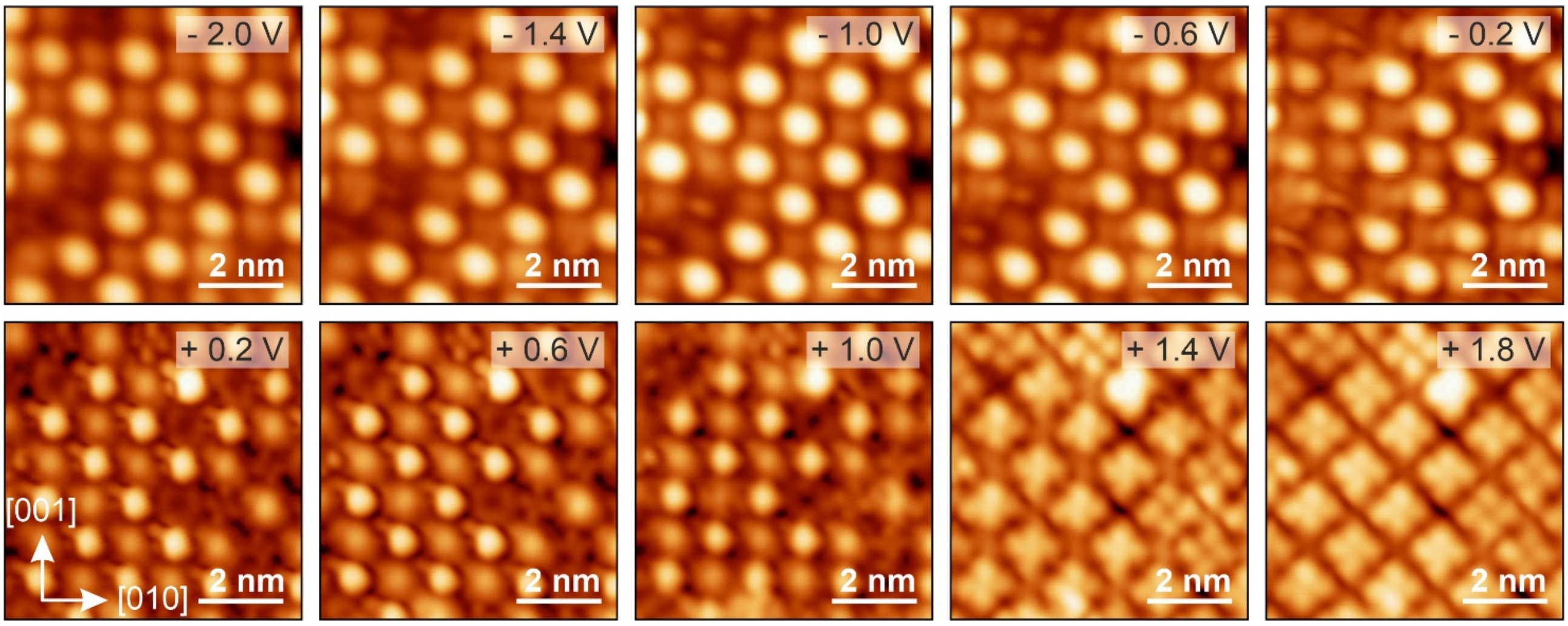

Figure S4. STM images of the MOF formed after deposition of 0.049 ML Co, acquired at different bias voltages as indicated by the insets. All images were recorded with a set tunneling current of 0.05 nA, except for the measurement at + 0.2 V, which was taken with a current of 0.02 nA. The image size for each measurement is 7 x 7 nm^2. The crystallographic directions for all images are indicated by arrows in the bottom left corner.

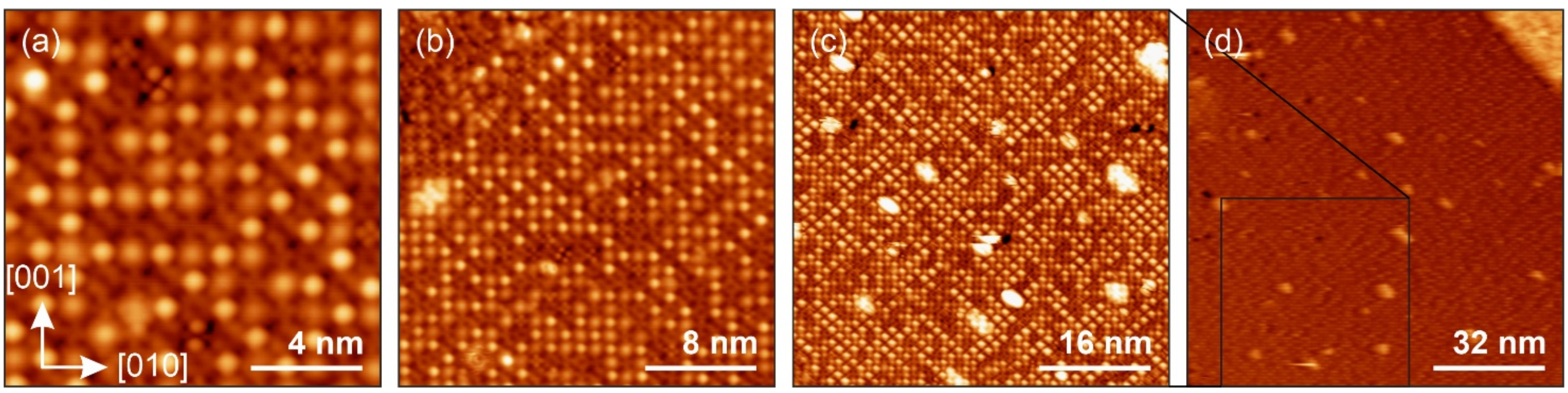

Figure S5. Large-scale STM images in different sizes ((a) 13.5 x 13.5 nm^2 (b) 27 x 27 nm^2 (c) 54 x 54 nm^2 (d) 108 x 108 nm^2) of the MOF formed after deposition of 0.044 ML Co. All images were acquired at a bias voltage of + 0.6 V, with a set tunneling current of 0.02 nA for (a) – (c) and 0.05 nA for (d). The crystallographic directions of the Au(100) surface, indicated by arrows in (a), apply to all images.

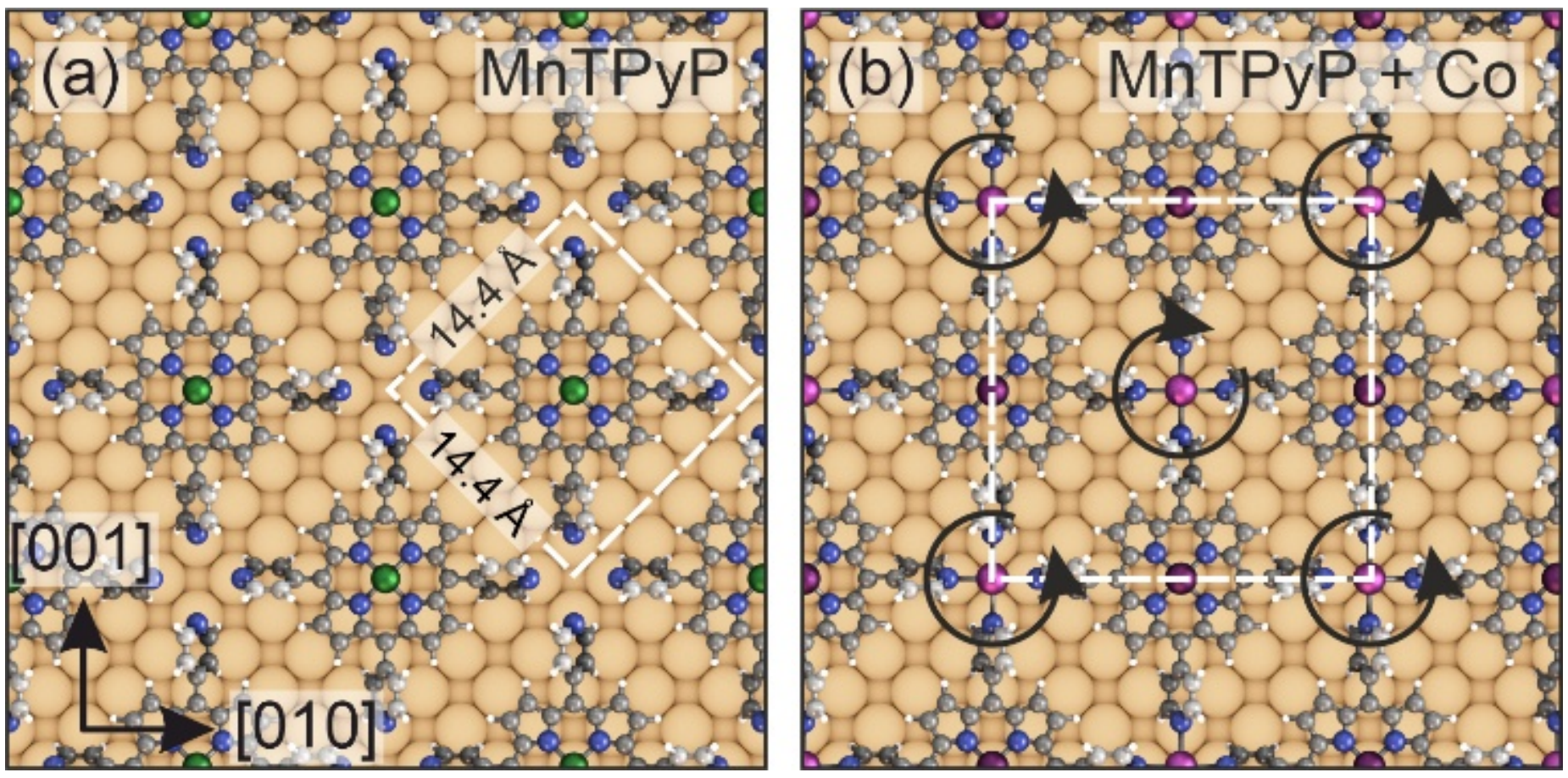

Figure S6. Real-space adsorption model of the self-assembled monolayer (a), showing one molecule per unit cell. Real-space adsorption model of the MOF (b). Due to alternating pyridyl rotations within each molecule, two molecules are in the unit cell of the MOF. Those models are derived from LEED and STM.

Section II – Removal of Chlorine upon annealing

After synthesis, the manganese atoms in the MnTPyP molecules are stabilized in the +3 oxidation state through coordination with a chloride ligand (Cl-MnTPyP). It is still under debate whether the Cl atom detaches from the metal center during the sublimation process and, if so, in which oxidation state the Mn is left upon chlorine removal.[15,16] As a first step, we investigated a powder sample of Cl-MnTPyP by XPS to assess whether Cl remains bound to the Mn atom upon mild annealing, and to evaluate how its removal affects the manganese oxidation state.

The Cl 2p and Mn $2p_{3/2}$ core level spectra of a thin layer of Cl-MnTPyP pressed onto carbon tape, before and after annealing at 420 K for 2 hours, are shown in **Figure S7**. Starting from the Cl 2p, the pristine film exhibits a broad peak centered at approximately 198 eV, which is associated with chlorine coordinated to the manganese center. The broadened appearance results from the spin-orbit splitting of the Cl 2p (the energy separation between the two components, namely $2p_{3/2}$ and $2p_{1/2,}$ is about 1 eV). Upon annealing, this peak nearly disappears, indicating that the thermal energy supplied to the system is sufficient to promote Cl detachment.

We now move to the Mn $2p_{3/2}$ core level spectra, which were fitted with a single Voigt function and a Shirley background. Before annealing (in **Figure S7b,** top), the Mn spectrum shows a single peak at 642.5 eV, typical for Mn in a +3 oxidation state.[17,18] After annealing (**Figure S7b,** bottom), the intensity shifts to 640.9 eV, concomitant with the reduction of Mn to the +2 oxidation state, resulting from the release of the chlorine.[19]

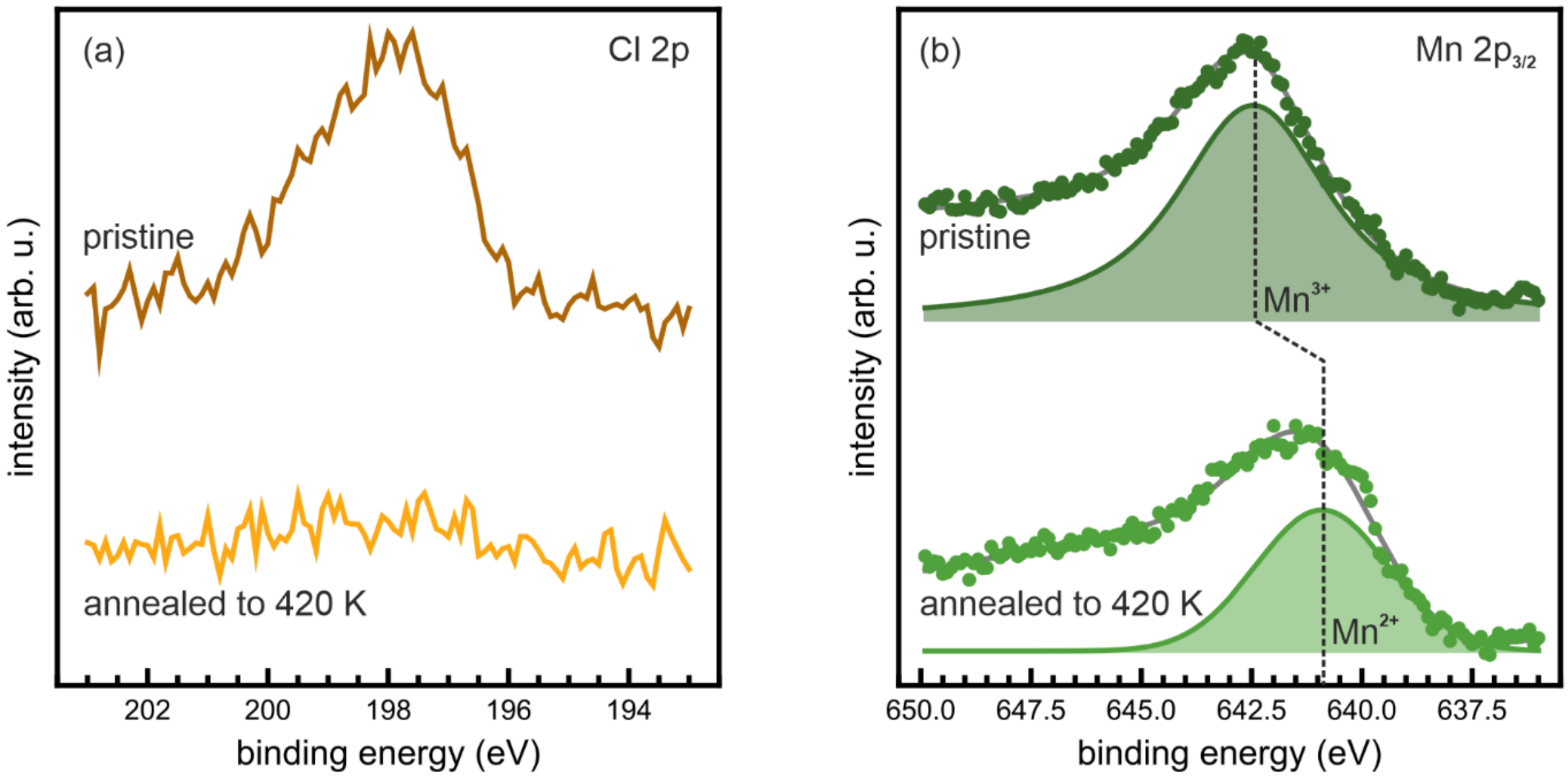

Figure S7. (a) XPS Cl 2p core level spectra and (b) XPS Mn $2p_{3/2}$ core level spectra of the powder Cl-MnTPyP sample before (brown and dark green, respectively) and after (yellow and light green, respectively) annealing at around 420 K.

As mentioned in the main text, the signal from the Mn 2p overlaps with the Au $4p_{1/2}$ for the molecular system atop gold. However, the presence of chloride ligands can still be evaluated by monitoring the Cl 2p core level spectrum of Cl-MnTPyP upon sublimation onto Au(100). In **Figure S8**, the spectra of the Cl 2p core level recorded before and after the deposition of a MnTPyP monolayer are shown. No significant changes are observed, indicating that chloride is no longer coordinated to the Mn center after deposition from the Knudsen cell onto the sample.

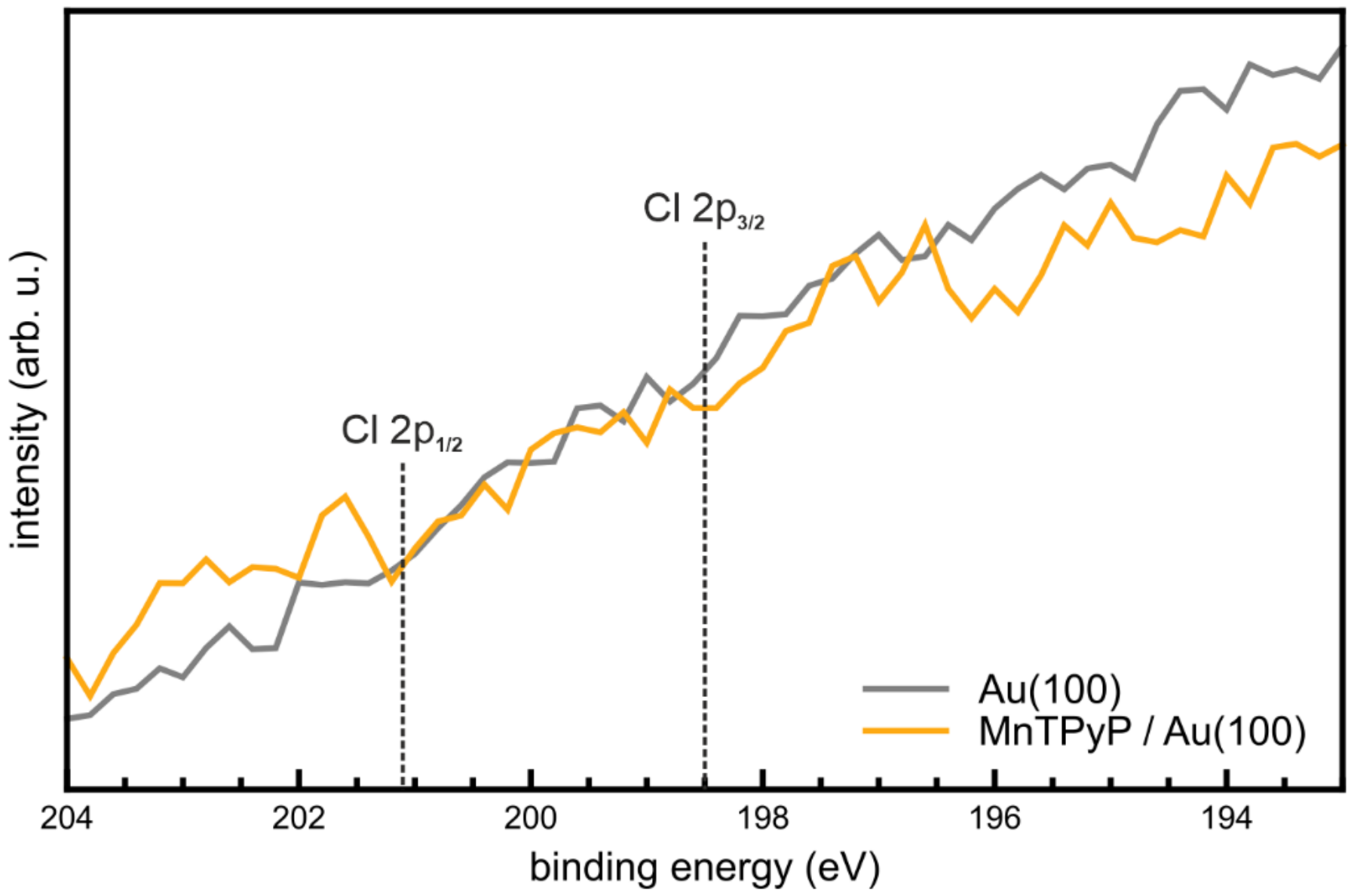

Figure S8. The Cl 2p core level spectra of the clean Au(100) (gray) and after depositing 2.4 saturated monolayers of MnTPyP (yellow).

Section III – N1s of the MnTPyP + Co

The MnTPyP molecules contain two non-equivalent nitrogen species: one in the macrocycle (which binds to the Mn) and the other at the pyridyl groups, which can be used to coordinate with an additional metal node, forming the metal-organic framework (MOF). For this reason, X-ray photoelectron spectroscopy (XPS) measurements of

the N 1s core level can provide additional information about the electronic structure of the macrocycle and the formation of the MOF upon deposition of Co.

The XPS N 1s spectrum acquired after the deposition of a saturated monolayer of MnTPyP on Au(100) is shown in the upper panel of **Figure S9**. In order to identify the contribution of the two non-equivalent nitrogen species, the spectrum was fitted using two Voigt curves combined with a Shirley background. To satisfy the stoichiometry of the molecules, the area ratio was fixed to 1:1. The two peaks were found at binding energies (BEs) of 397.9 eV and 398.6 eV, respectively. By comparing our data with existing references, we found that the peak at lower BE originates from the pyrrolic nitrogen,[1] while the other one belongs to the uncoordinated pyridinic nitrogen.[2–4]

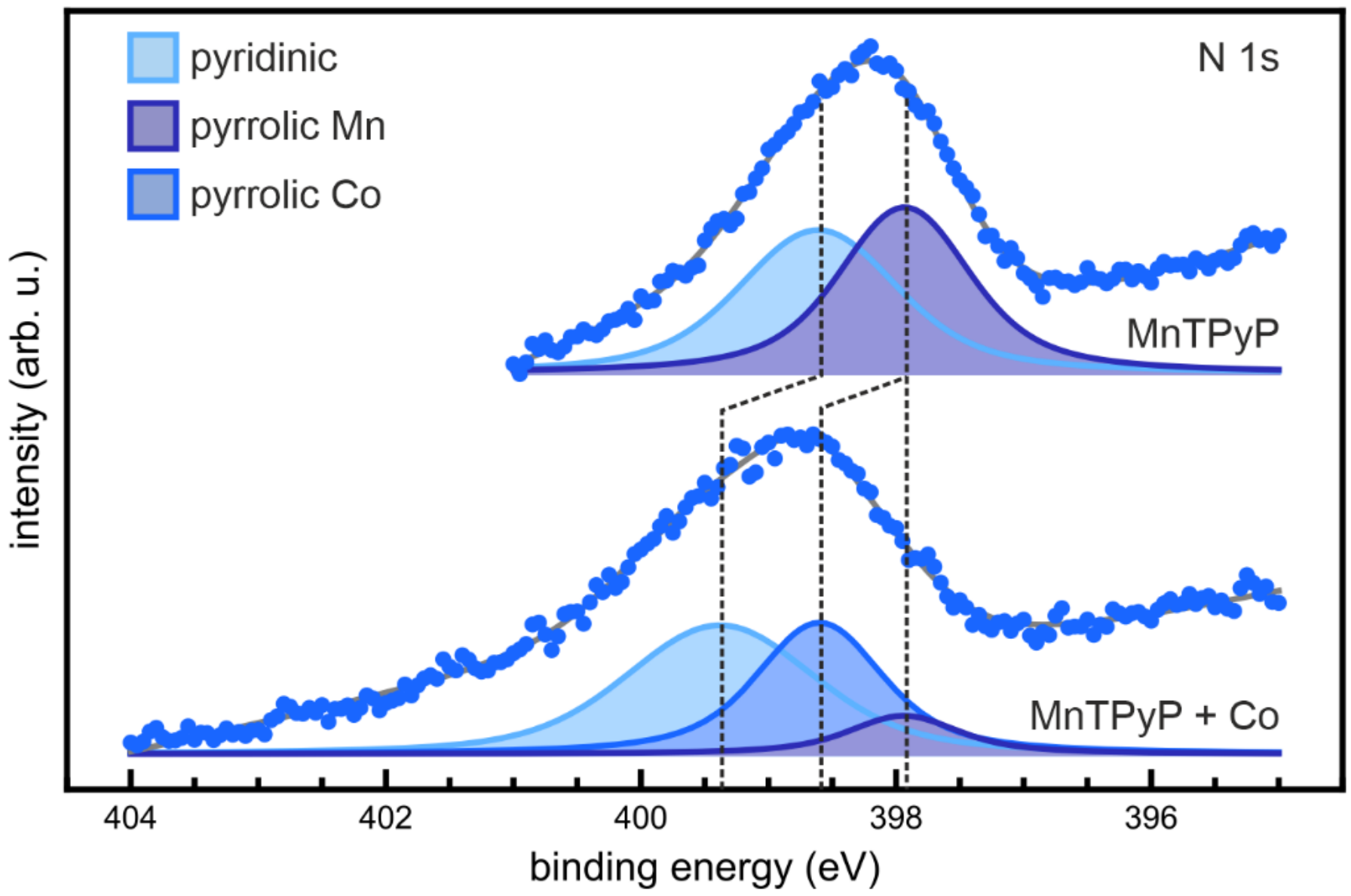

Figure S9. XPS N 1*s* core level spectra of the molecular layer of MnTPyP on Au(100) (top) and of the MOF formed upon deposition of 0.072 ML cobalt (bottom), showing that coordination of the cobalt with the pyridyl is confirmed by a shift of the intensity towards higher binding energy.

After the deposition of cobalt (0.072 ML) and the formation of the MOF, the overall lineshape of the spectrum changes (see lower panel of **Figure S9**). The spectrum can be fitted using three components centered at 397.9 eV, 398.6 eV, and 399.4 eV, respectively. To assign each peak to a specific nitrogen species, we performed a thorough comparison with the literature and a control experiment. The control XPS experiment consists of a molecular layer of CoTPyP, the final result of the proposed transmetallation, and of a homometallic network Co–CoTPyP on Au(100), described in detail in Section V. Our analysis reveals that the peak associated with the pyridinic nitrogen shifts to a binding energy of 399.4 eV.[5] This assignment is further confirmed by our control experiment, where the component associated with the Co-coordinated pyridyl moieties is found at the same BE. The transmetallation process affects the energy position of the pyrrolic peak, found at 398.6 eV. Similar values are observed for CoTPyP deposited on graphene upon the formation of the homometallic Co-CoTPyP MOF[5] and in our control experiment. This feature can still also exhibit a small contribution of uncoordinated pyridinic nitrogen, found at the same BE for the pristine molecular layer. Further confirmed by remaining intensity at 397.9 eV, attributed to pyrrolic nitrogen still coordinated with Mn, and the required 0.008 ML more cobalt for a fully saturated MOF layer.

Table S1. Fitting parameters for the N 1s core level. The Lorentzian and Gaussian parameters describe the corresponding FWHM. All the values are given in eV, except for the areas, which are in arbitrary units.

	MnTPyP	MnTPyP + Co	CoTPyP	Co-CoTPyP
**Lorentzian**	0.68	0.68	0.68	0.68
**Pyrrolic Mn energy pos.**	397.9	397.9 (fixed)	-	-
**Pyrrolic Mn area**	250	50	-	-
**Pyrrolic Mn Gaussian**	0.80	0.58	-	-
**Pyrrolic Co energy pos.**	-	398.6 (fixed)	398.3	398.6
**Pyrrolic Co area**	-	191	210	178
**Pyrrolic Co Gaussian**	-	0.74	0.58	0.58
**Pyridinic energy pos.**	398.6	399.4 (fixed)	398.4	399.4
**Pyridinic area**	250	260	210	178
**Pyridinic Gaussian**	1.06	1.36	0.62	1.24

Section IV – Co L-edge

In **Figure S10**, the NEXAFS spectra recorded at the Co L_3-edge for both s- and p-polarized light after MOF formation are shown. The spectra exhibit two well-resolved resonances: one at 776.8 eV (A), most pronounced in p-polarization, and another at 778.6 eV (B), which is mainly visible in s-polarization. The presence of a pronounced dichroic signal confirms that the Co centers are embedded in a well-defined ligand field, consistent with MOF formation and the substitution of Mn by Co in the porphyrin macrocycle. We note that, because of the problem in the absolute photon energy calibration, as described in the experimental part, there still could be a discrepancy with literature values (investigation of similar systems leads, sometimes, to a discrepancy in the order of approximately 2 eV).[6,20] Therefore, instead of comparing the absolute energy position of the resonance with reference values, we focus on the dichroism of each resonance and its relative energy positions. Because of

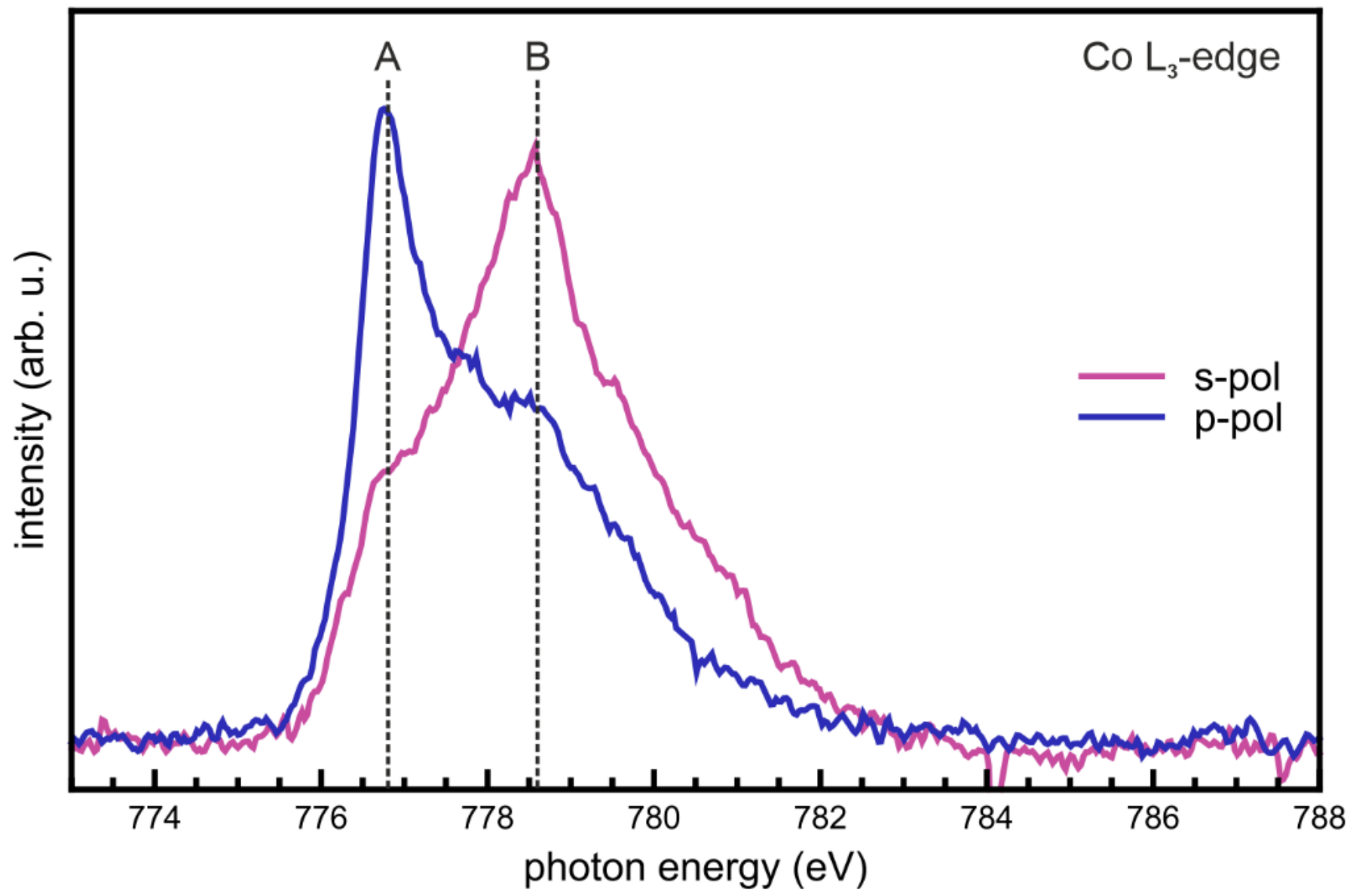

Figure S10. The Co L_3-edge spectrum of the network layer, showing a multiplet structure, which confirms its coordination.

the presence of two Co species with different oxidation states and, thus, a multiplet structure, it is difficult to assign each resonance to either one or the other cobalt atom. Thus, in the following, we will tentatively assign each transition to a specific *d* orbital, rather than speculate from which Co species it originates.

According to Zhang et al., who conducted a joint gas-phase and solid-state photoemission and absorption spectroscopy study, supported by DFT calculations,[21] resonance A is associated with the transitions from the $2p_{3/2}$ level into the d_{z2} orbital, which possesses out-of-plane symmetry. This is consistent with the fact that this resonance mainly appears when exciting the system with p-polarized light. Resonance B, instead, corresponds to the excitation into the d_{x2y2} orbitals. As established by our own DFT calculations, for the Co chelated in the macrocycle the d_{z2} orbital is half-filled and the d_{x2y2} orbital is empty (compare to **Figure S15**). Therefore, this Co(II) species contributes to both resonances. In case of a simplified picture of the Co(I) species, it would be expected that the d_{z2} orbital is filled entirely whereas the d_{x2y2} orbital remains empty. However, based on our calculations the d_{z2} and the d_{x2y2} of the Co(I) species mix (see Figure 5 in the main text). For this purpose, we refrain from assigning either one of the resonances to one of the two cobalt species.

Section V – The control experiment: Co-CoTPyP homometallic framework

As a control experiment, a single layer of CoTPyP sublimated on Au(100) was characterized with low-energy electron diffraction (LEED) and XPS before and after the deposition of Co and the resulting the formation of a homometallic Co-CoTPyP MOF.

Geometric arrangement

The LEED of a saturated monolayer of CoTPyP/Au(100) is reported in **Figure S11a**. The resulting pattern resembles that of the self-assembled MnTPyP film (see **Figure 1a** in the main text), suggesting that the final unit cell is the same for both molecules. A similar situation is found after depositing Co on the CoTPyP/Au(100) interface: the resulting diffraction pattern in **Figure S11b** shows the same periodicity as the pattern obtained after depositing Co on the MnTPyP film (see **Figure 1d** in the main text), pointing also in this case to the same geometric arrangement shown in **Figure 1f**, in the main text.

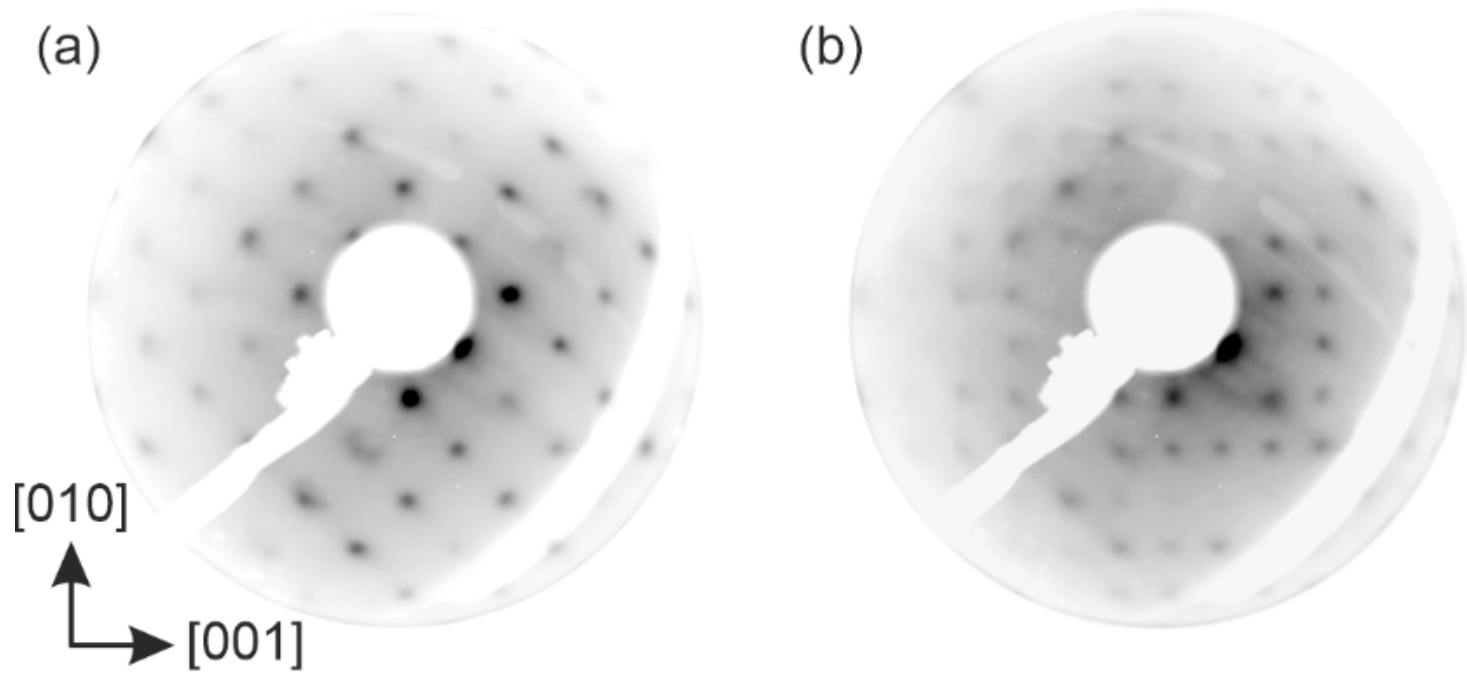

Figure S11. LEED measurements at an electron kinetic energy of 25 eV of (a) the molecular layer CoTPyP, and (b) the Co-CoTPyP MOF on Au(100).

N 1s before and after the formation of the Co-CoTPyP MOF

Like MnTPyP, the CoTPyP contains two non-equivalent nitrogen species. To comply with the stoichiometry of the molecules, the N 1s spectrum of CoTPyP atop Au(100) in **Figure S12** (top panel) was fitted with two Voigt functions with an area ratio of 1:1. This yields the two peaks at 398.3 eV and 398.4 eV, respectively. Since these two peaks overlap in energy, we cannot make a clear assignment based on our data. However, after the deposition of cobalt, the N 1s lineshape changes clearly, and one of the two components shifts to 399.4 eV (light blue peak in the lower panel of **Figure S12**). Since no transmetallation is expected here, and therefore no change on the feature assigned to the pyrrolic nitrogen, the shifting contribution is associated with the pyridinic nitrogen, as it is the species that changes from uncoordinated to coordinated with the additional Co atom, leading to the formation of the MOF.

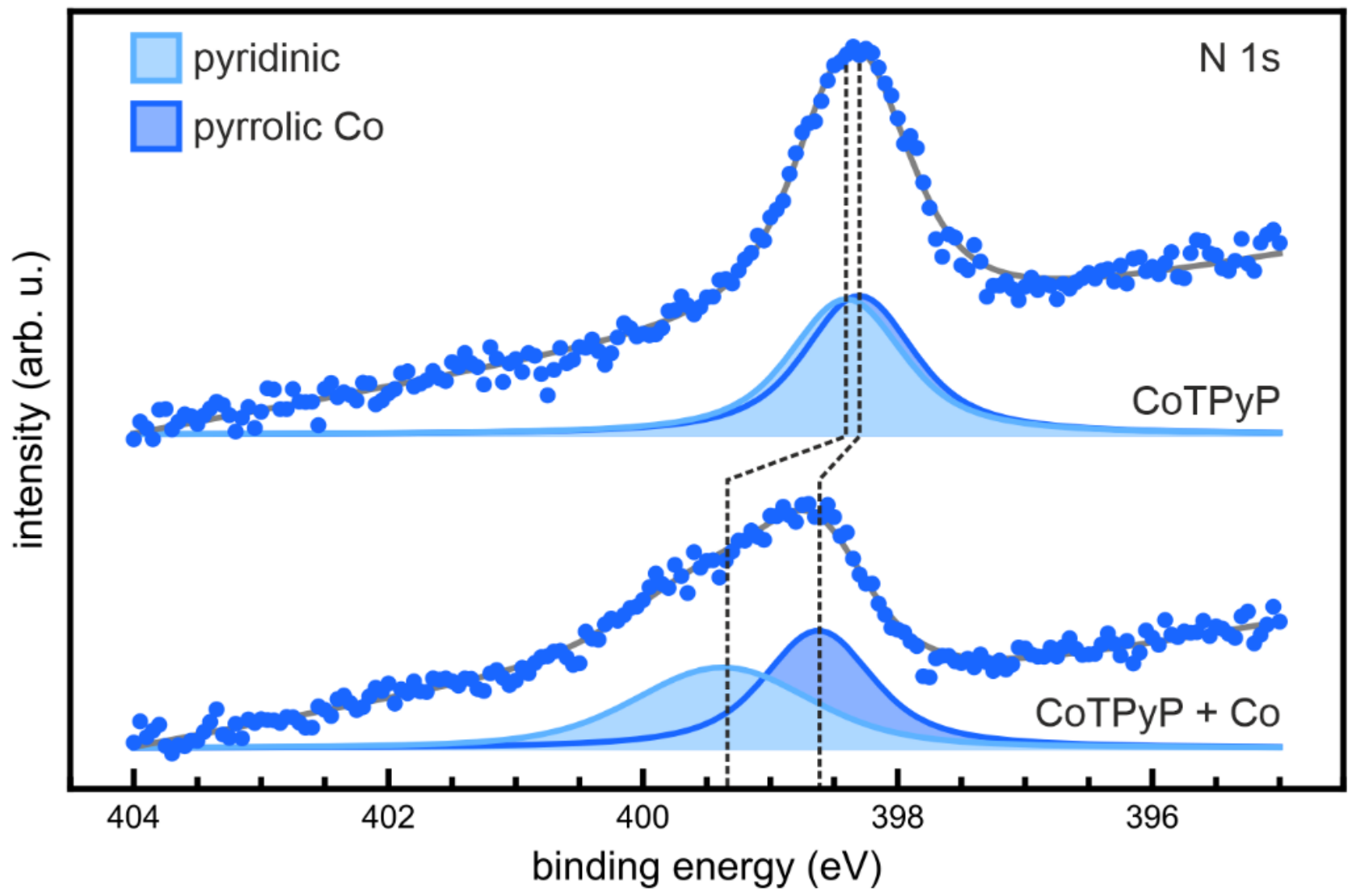

Figure S12. XPS N 1s core level spectra of the molecular layer of CoTPyP on Au(100) (top) and of the MOF formed upon cobalt deposition (bottom).

Co 2p$_{3/2}$ before and after the formation of the Co-CoTPyP MOF

The Co 2p$_{3/2}$ core level spectrum of a CoTPyP saturated monolayer deposited on Au(100), before and after the additional deposition of Co atoms, is shown in **Figure S13**. The pristine CoTPyP film (top panel) exhibits a single peak, which is fitted using a single Voigt function combined with a Shirley background. The most important fit parameters are listed in **Table S2**. From the fit, the binding energy (BE) of this component is determined to be 779.7 eV. We attribute this peak to the Co inside the macrocycle in a +2 oxidation state, consistent with other Co-containing porphyrins[6,7] and phthalocyanines.[8] A satellite at a higher BE arises due the multiplet splitting originating from the unpaired electron in the +2 oxidation state (d^7).[6,7,9] It is worth pointing out that we restrict all peaks to share the same Lorentzian width. The relatively broad energy resolution provided by the lab X-ray source leads to several fitting solutions. It is thus convenient to reduce the number of fitting parameters to avoid overfitting.

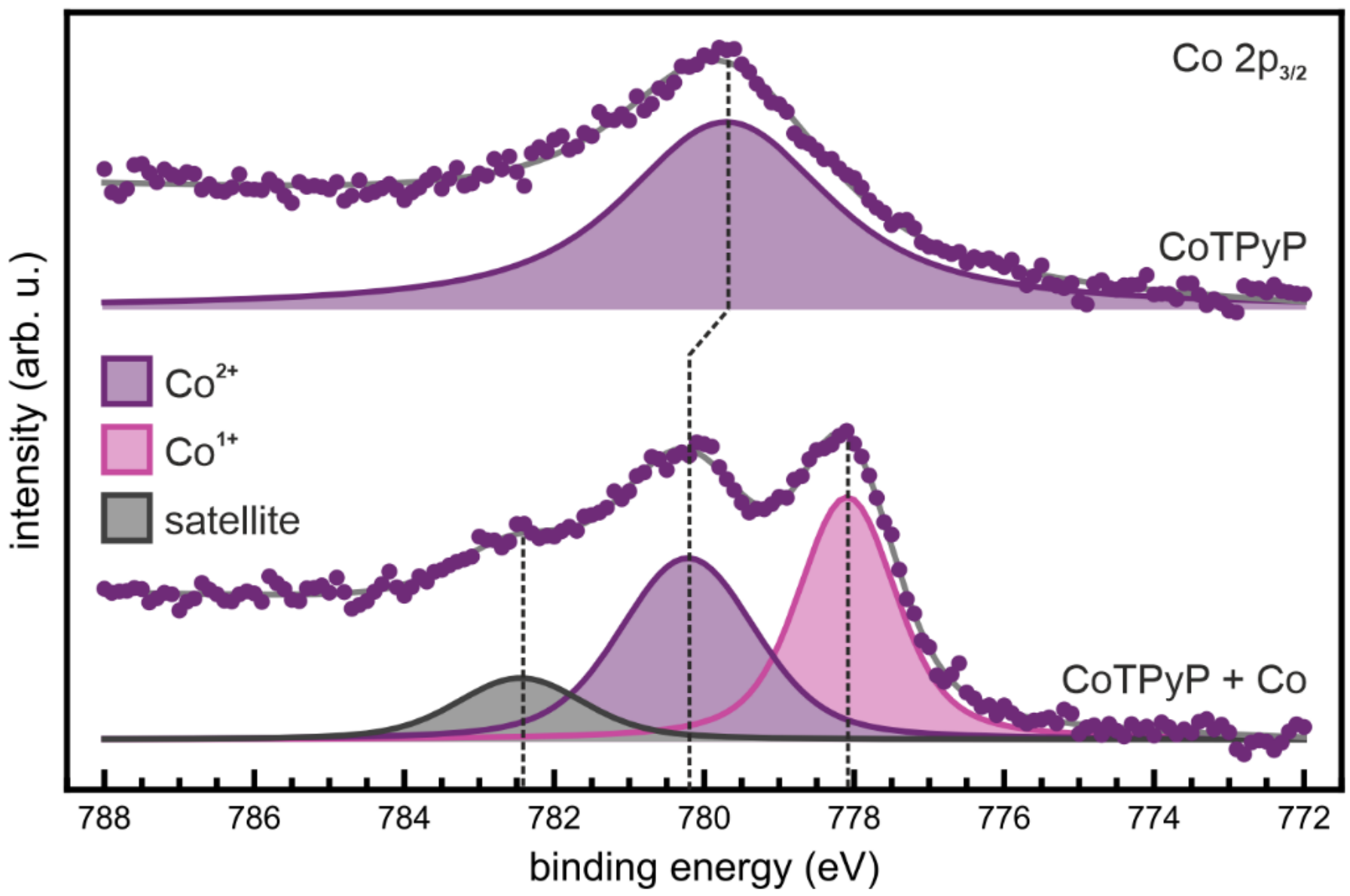

Figure S13. XPS Co 2p$_{3/2}$ core level spectra of the molecular layer of CoTPyP on Au(100) (top) and of the MOF formed upon cobalt deposition (bottom).

The Co deposition leads to an overall spectrum change: the peak originating from the Co centered in the macrocycle shifts slightly toward a higher binding energy of 780.2 eV. In addition, a new prominent peak appears at 778.1 eV (light purple), and a shoulder attributed to the unresolved multiplet and satellite structure emerges

(gray). This new prominent peak is associated with the second Co atom that coordinates within the pyridyl moieties forming the MOF, as shown by the analysis of both the NEXAFS measurements at the N K-edge and the XPS N 1*s* spectra. Although the energy position of this peak is close to that of metallic Co[10], a comparison with literature reveals that this Co species is in the +1 oxidation state.[6,11–13] More specifically, the main Co $2p_{3/2}$ resonance is found at a similar BE for both CoTPP/Ag(111) and CoTPP/Cu(100), where the Co is reduced due to charge transfer from the substrate. Further support comes from density functional theory calculations.[6] This +1 oxidation state can also be found for Co-CoTPyP and Co-MnTPyP MOF synthesized atop graphene.[11,12,14] Thus, the additional Co, although coordinated with four nitrogen atoms in a ligand field — similarly to the Co atom in the macrocycle — does not exhibit the same oxidation state.

Table S2. Fitting parameters for the Co $2p_{3/2}$ core level. The Lorentzian and Gaussian parameters describe the corresponding FWHM. All the values are given in eV, except for the areas, which are in arbitrary units.

	CoTPyP	Co-CoTPyP	MnTPyP + Co
**Lorentzian**	2.61	0.71	1.43
**Co^{1+} energy pos.**	-	778.1	778.0
**Co^{1+} area**	-	870	1153
**Co^{1+} Gaussian**	-	1.11	0.86
**Co^{2+} energy pos.**	779.7	780.2	780.2
**Co^{2+} area**	1612	857	930
**Co^{2+} Gaussian**	1.43	1.73	2.13
**Satellite energy pos.**	-	782.5	782.5 (fixed)
**Satellite area**	-	275	165
**Satellite Gaussian**	-	1.59	1.36

Section VI – Co coverage

In **Figure S14,** we report a series of XPS spectra taken at the Co $2p_{3/2}$ core level for different Co coverages on a saturated monolayer of MnTPyP atop Au(100). In Section V we already assigned the two distinct peaks to either Co(I) (lower BE) or Co(II) (higher BE). The former contribution results from the MOF formation, while the latter originates from the Co atom within the porphyrin macrocycle upon transmetallation. Therefore, we can use this measurement series to assess whether the two processes (MOF formation vs transmetallation) occur simultaneously.

Up to a coverage of 0.67 ML of Co, the spectra exhibit similar lineshapes, with the two peaks growing by the same amount after each evaporation step. Please note that for a saturated MnTPyP layer, 0.08 ML of Co corresponds to the coverage for which all molecules are transmetallated and coordinated. Therefore, these measurements point towards the simultaneous occurrence of transmetallation and coordination of the MOF. Above 0.08 ML of Co, the spectral shape changes. Because this coverage exceeds full saturation, metallic cobalt clusters begin to form, which in turn explains the differences.

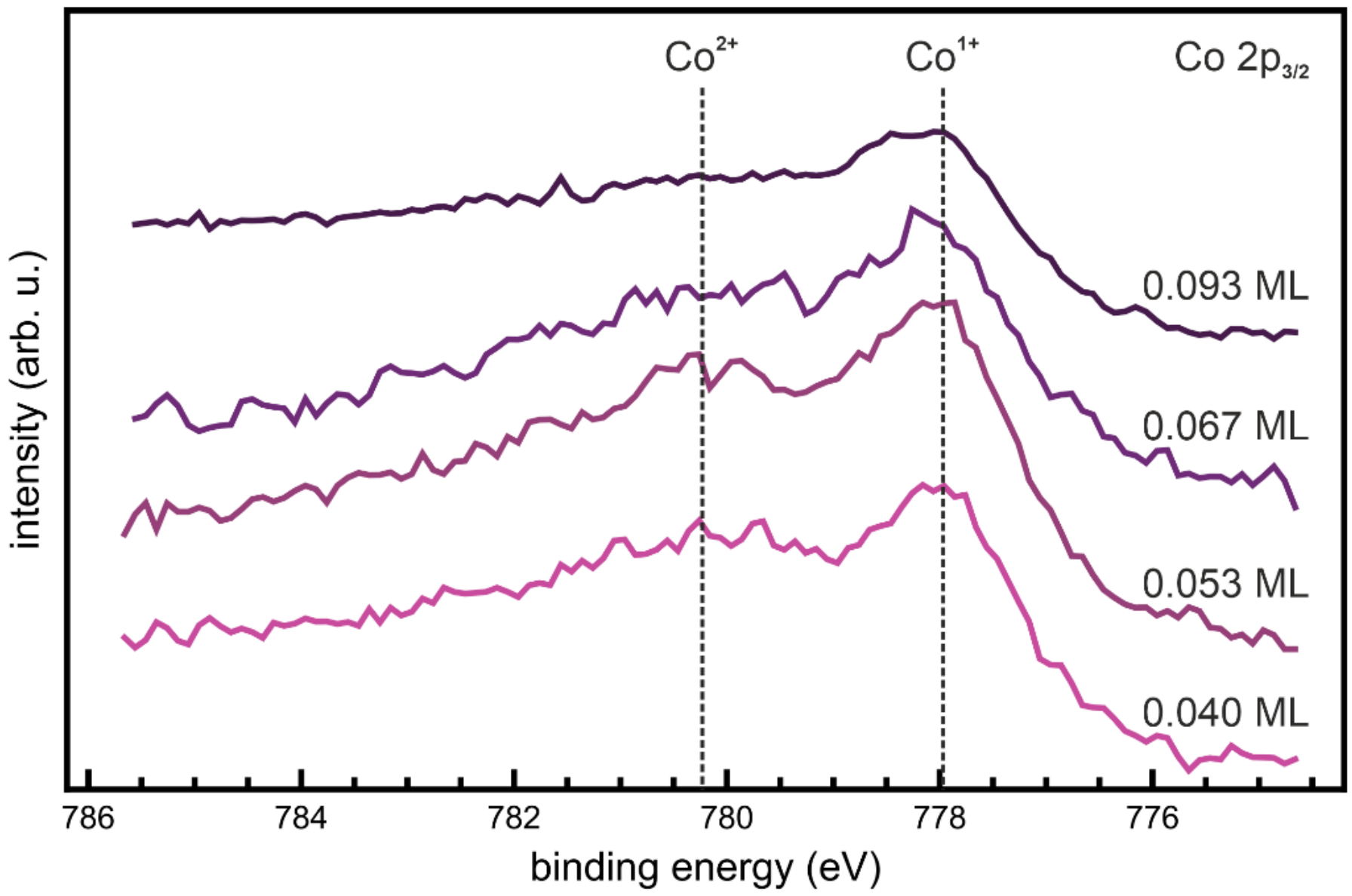

Figure S14. XPS Co $2p_{3/2}$ core level spectra of different Co coverages atop a saturated monolayer of MnTPyP on Au(100). The simultaneous growth of the two peaks indicates that coordination and transmetallation occur at the same time.

Section VII: DOS macrocycle

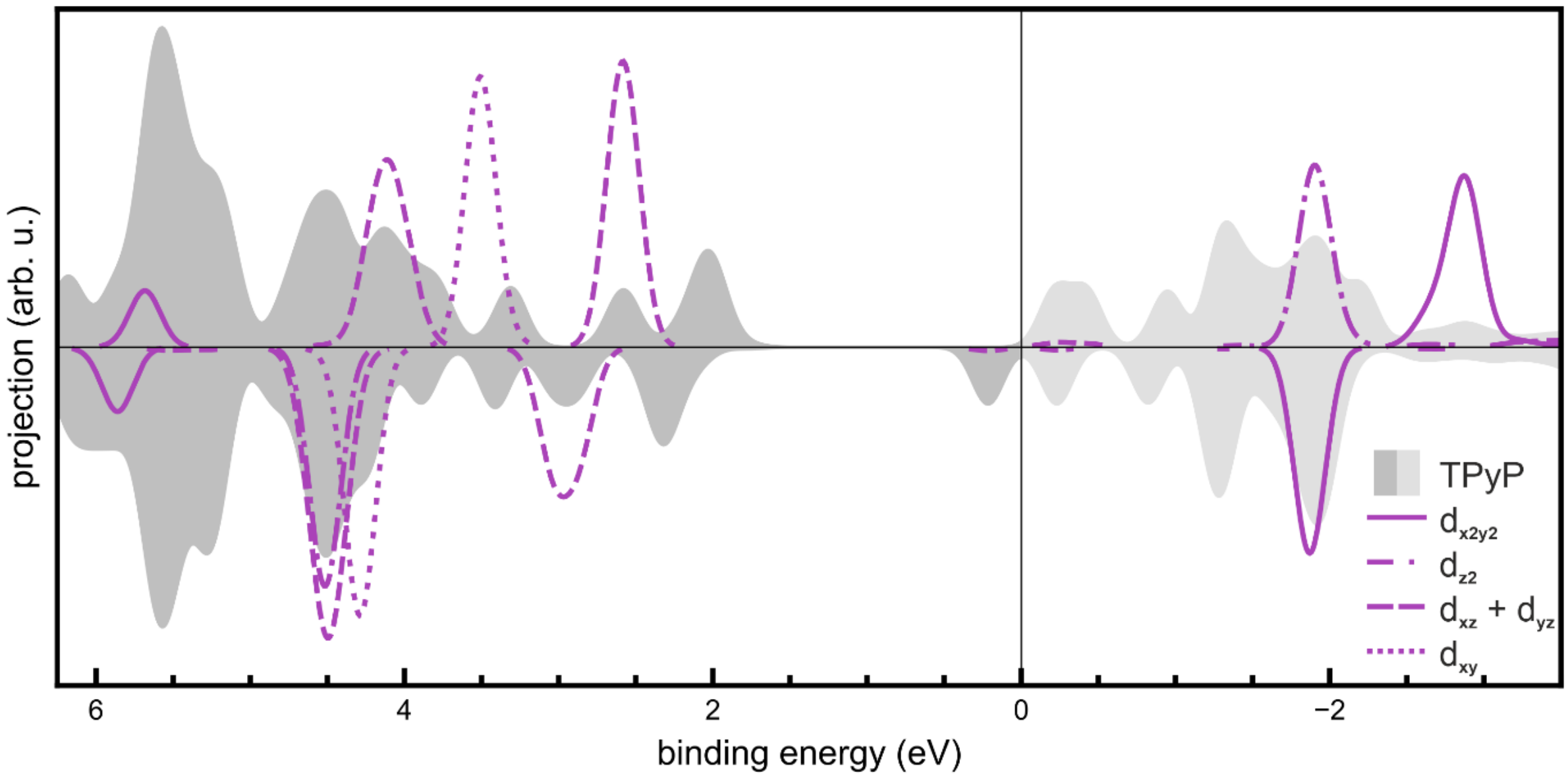

Figure S15. Density of states for a substrate free transmetallated Co-CoTPyP projected onto the 3d states of the Co in the porphyrin macrocycle (purple lines) and atomic projections onto the N, C and H atoms (gray shaded area) of the TPyP molecule.

References

1. Zhang, J. L. *et al.* Single-Molecule Imaging of Activated Nitrogen Adsorption on Individual Manganese Phthalocyanine. *Nano Lett.* **15**, 3181–3188 (2015).
2. Chen, M. *et al.* Direct Synthesis of Nickel(II) Tetraphenylporphyrin and Its Interaction with a Au(111) Surface: A Comprehensive Study. *J. Phys. Chem. C* **114**, 9908–9916 (2010).
3. Yang, H. B. *et al.* Atomically dispersed Ni(I) as the active site for electrochemical CO2 reduction. *Nat Energy* **3**, 140–147 (2018).
4. Li, Y. *et al.* Coordination and Metalation Bifunctionality of Cu with 5,10,15,20-Tetra(4-pyridyl)porphyrin: Toward a Mixed-Valence Two-Dimensional Coordination Network. *J. Am. Chem. Soc.* **134**, 6401–6408 (2012).
5. Armillotta, F. *et al.* Spectroscopic fingerprints of iron-coordinated cobalt and iron porphyrin layers on graphene. *CR-PHYS-SC* **4**, (2023).
6. Cojocariu, I. *et al.* Reversible redox reactions in metal-supported porphyrin: the role of spin and oxidation state. *J. Mater. Chem. C* **9**, 12559–12565 (2021).
7. Lukasczyk, T. *et al.* Interaction of Cobalt(II) Tetraarylporphyrins with a Ag(111) Surface Studied with Photoelectron Spectroscopy. *J. Phys. Chem. C* **111**, 3090–3098 (2007).
8. Schmid, M., Kaftan, A., Steinrück, H.-P. & Gottfried, J. M. The electronic structure of cobalt(II) phthalocyanine adsorbed on Ag(111). *Surface Science* **606**, 945–949 (2012).
9. Hieringer, W. *et al.* The Surface Trans Effect: Influence of Axial Ligands on the Surface Chemical Bonds of Adsorbed Metalloporphyrins. *J. Am. Chem. Soc.* **133**, 6206–6222 (2011).
10. Biesinger, M. C. *et al.* Resolving surface chemical states in XPS analysis of first row transition metals, oxides and hydroxides: Cr, Mn, Fe, Co and Ni. *Applied Surface Science* **257**, 2717–2730 (2011).
11. Armillotta, F. *et al.* Co(III), Co(II), Co(I): Tuning Single Cobalt Metal Atom Oxidation States in a 2D Coordination Network. *Adv Funct Materials* 2408200 (2024) doi:10.1002/adfm.202408200.
12. Bidoggia, D., Armillotta, F., Sala, A., Vesselli, E. & Peressi, M. Site-Dependent Oxidation States of Single Cobalt Atoms in a Porphyrin-Based Monolayer on Graphene. *J. Phys. Chem. C* **128**, 1737–1745 (2024).

13. Baronio, S. Reactivity of porphyrin- and phthalocyanine-based assemblies at surfaces from UHV to near-ambient pressure. (Università degli studi di Trieste, Trieste, 2025).

14. Baronio, S. *et al.* Single atom coordination in a manganese–cobalt bi-metallic framework on graphene: geometric and electronic structures. *Nanoscale* https://doi.org/10.1039/D5NR01383F (2025) doi:10.1039/D5NR01383F.

15. Murphy, B. E. *et al.* Homolytic Cleavage of Molecular Oxygen by Manganese Porphyrins Supported on Ag(111). *ACS Nano* **8**, 5190–5198 (2014).

16. De Col, M. *et al.* Carbon monoxide ligation at single metal atom sites in a 2D manganese-cobalt coordination network: equilibrium at room temperature. *J. Mater. Chem. A* 10.1039.D5TA10405J (2026) doi:10.1039/D5TA10405J.

17. Sugiura, K. *et al.* Synthesis, properties, molecular structure and electron transfer salts of 13,13,14,14-tetracyano-1,6- and -1,8-pyrenoquinodimethanes (1,6-TCNP and 1,8-TCNP). *J. Mater. Chem.* **10**, 315–319 (2000).

18. Töpfer, J. *et al.* Cation Valencies and Distribution in the Spinels NiMn2O4 and MzNiMn2−zO4 (M = Li, Cu) Studied by XPS. *physica status solidi (a)* **134**, 405–415 (1992).

19. Allen, G. C., Harris, S. J., Jutson, J. A. & Dyke, J. M. A study of a number of mixed transition metal oxide spinels using X-ray photoelectron spectroscopy. *Applied Surface Science* **37**, 111–134 (1989).

20. Hötger, D. *et al.* On-surface transmetalation of metalloporphyrins. *Nanoscale* **10**, 21116–21122 (2018).

21. Zhang, T. *et al.* Conclusively Addressing the CoPc Electronic Structure: A Joint Gas-Phase and Solid-State Photoemission and Absorption Spectroscopy Study. *J. Phys. Chem. C* **121**, 26372–26378 (2017).
